# Bridging the gap between efficacy trials and model-based impact evaluation for new tuberculosis vaccines

**DOI:** 10.1038/s41467-019-13387-9

**Published:** 2019-11-29

**Authors:** Mario Tovar, Sergio Arregui, Dessislava Marinova, Carlos Martín, Joaquín Sanz, Yamir Moreno

**Affiliations:** 1 Institute for Bio-computation and Physics of Complex Systems (BIFI), University of Zaragoza, Zaragoza, Spain; 2 Department of Theoretical Physics, Faculty of Sciences, University of Zaragoza, Zaragoza, Spain; 3 Microbiology Department, Faculty of Medicine, University of Zaragoza, Zaragoza, Spain; 4 Networked Biomedical Research Center on Respiratory Disease CIBERES, Madrid, Spain; 5 Service of Microbiology, Miguel Servet Hospital, IIS Aragon, Zaragoza, Spain; 6 Department of Medicine, Genetics Section, University of Chicago, Chicago, IL USA; 7 ISI Foundation, Turin, Italy

**Keywords:** Computational models, Vaccines, Tuberculosis, Randomized controlled trials

## Abstract

In Tuberculosis (TB), given the complexity of its transmission dynamics, observations of reduced epidemiological risk associated with preventive interventions can be difficult to translate into mechanistic interpretations. Specifically, in clinical trials of vaccine efficacy, a readout of protection against TB disease can be mapped to multiple dynamical mechanisms, an issue that has been overlooked so far. Here, we describe this limitation and its effect on model-based evaluations of vaccine impact. Furthermore, we propose a methodology to analyze efficacy trials that circumvents it, leveraging a combination of compartmental models and stochastic simulations. Using our approach, we can disentangle the different possible mechanisms of action underlying vaccine protection effects against TB, conditioned to trial design, size, and duration. Our results unlock a deeper interpretation of the data emanating from efficacy trials of TB vaccines, which renders them more interpretable in terms of transmission models and translates into explicit recommendations for vaccine developers.

## Abstract (web summary)

One measurement of tuberculosis vaccine efficacy in clinical trials is prevention of disease, but different mechanisms can underlie disease prevention. Here, the authors develop a mathematical model that allows to identify mechanisms of action of a vaccine preventing TB disease.

## Introduction

Despite the global decline in tuberculosis (TB) burden during the present century, it still remains one of the greatest threats to public health worldwide. According to the last Global TB Report of the World Health Organization (WHO)^1^, 10 million people developed TB during 2018 and 1.45 million people were killed by it. Furthermore, the impact of HIV–TB co-infection^2,3^, accounting for 251,000 of these deaths^1^, and the emergence of multi- and extensively drug-resistant TB strains^4,5^ is devastating. These realities point to the pressing need for the development of new control methods and epidemiological interventions, namely the introduction of a new vaccine. As of today, the research community is engaged in pursuing many different candidates for a new TB vaccine, 12 of which are being tested in clinical trials^1^.

However, the development of TB vaccines is plagued with many conceptual challenges that make difficult the evaluation of different candidates across the clinical pipeline^6^. The lack of protection correlates for TB^7,8^ hinders early efficacy evaluations, which forces researchers to wait until late stages, typically phases 2b and 3 of the clinical pipeline to assess vaccine efficacy. These trials require the recruitment and monitoring of thousands of individuals in high TB incidence settings during several years. In this regard, the neatly designed phase 2b trial of the MVA85A vaccine advanced a solid quantitative framework for defining minimum cohort sizes and follow-up periods in contemporary epidemiological settings, even though it failed to provide evidence of significant protection^9^. More studies have followed its steps, including different types of phase 2b trials for other vaccines, such as H4:IC31^10^ and the M72/AS01E^11^, which showed 54% efficacy ($95 \%$ CI: 2.9–78.2$\%$) against active TB in adult individuals already exposed to *Mycobacterium tuberculosis* (*M.tb*).

Phase 2b clinical trials can be designed to estimate different types of vaccine efficacy, including prevention of infection (POI), prevention of disease (POD) and prevention of recurrence (POR)^12^. Once these effects have been estimated for a given vaccine, its potential impact is estimated through the use of mathematical models of pathogen transmission^13^. However, trial-derived readouts of vaccine efficacy do not always guarantee an unequivocal interpretation from a TB modelling perspective. This is due to the extreme complexity of the natural history of the disease, which enables different dynamic mechanisms through which a vaccine can provide protection.

**Fig. 1:**
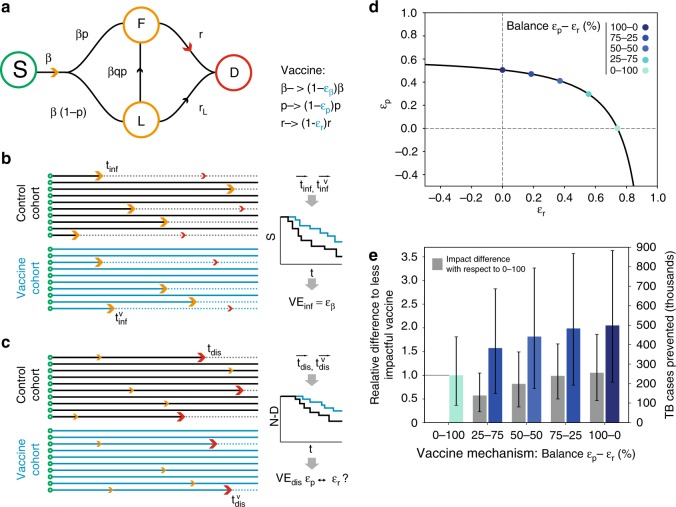
Equal prevention readouts from vaccine efficacy trials can map to multiple vaccine mechanisms and expected vaccine impacts. **a** Elementary *M.tb*. transmission model. S=susceptible, F=infected, fast progression to disease, L=infected, slow progression to disease (LTBI), D=active TB. The epidemiological parameters (black) can be modified by the vaccine effects (blue). **b** From the distributions of transition times between the beginning of the trial (green dots) until end-point infection (orange arrows), survival curves are built for the control and vaccine cohorts, and from their analysis, ${\text{VE}}_{\text{inf}}$ is estimated. **c** Equivalent schematics for the estimation of ${\text{VE}}_{\text{dis}}$ from survival analysis of transition times from trial’s beginning (green) to the end point associated with active TB (red arrows). **d** Curve of values of (${\varepsilon }_{{\rm{p}}}$,${\varepsilon }_{{\rm{r}}}$) compatible with a measurement of POD of ${\text{VE}}_{\text{dis}}=0.5$ after 4 years of follow-up (assuming no POI, i.e. ${\varepsilon }_{\upbeta }=0$). We have marked five different points in this curve, with different balances between ${\varepsilon }_{{\rm{p}}}$ and ${\varepsilon }_{{\rm{r}}}$, to be used in the next example. **e** Foreseen impacts obtained after introducing the vaccines highlighted in **d** in Ethiopia, at the end of 2025. Blue bars: vaccine impacts. Grey bars: difference in impact estimated between each vaccine and the least impactful case of a vaccine acting enterily through ${\varepsilon }_{{\rm{r}}}$ (lightest blue). Impacts are estimated by using a large-scale transmission model^16^ as the number of TB cases prevented in the country by the vaccine during the period 2026–2050. Error bars (black bars) represent the 95% confidence interval.

After an initial infection with *M.tb*, the causative agent of TB, some individuals develop TB within incubation periods of less than $\simeq$2 years^14^ (i.e. fast progression). On the other hand, others succeed at containing the infection and become asymptomatic, latent TB-infected individuals (LTBI). LTBI subjects remain so often for the rest of their lives, although they can suffer endogenous reactivation of the disease, even decades after the first infection event. Finally, they can also be re-infected and progress quickly to TB only after the secondary infection. These three possible routes to disease can be described as sketched in Fig. 1a^13,15,16^, which arguably reflects one of the most elementary model architectures, among the different options that can be used to describe the initial stages of the transmission chain of *M.tb*. that include a description of the incubation period of fast progression, a key ingredient for our analyses^17^.

From this outlook, a vaccine can confer POD through different mechanisms. On the one hand, it may act by decreasing the fraction of individuals that undergo fast progression to TB after an infection—or reinfection—event. However, it is also possible that the vaccine delays the onset of active TB, slowing down the dynamics of fast progression instead of preventing it. These two possible mechanisms have different dynamical interpretations, and in principle, may appear independent and not necessarily correlated. Factors that affect the probability of an individual to develop a fast or slow path to disease are environmental and genetic. However, little is known about whether or how they impact the delay observed between infection and onset of symptoms in TB cases associated with recent transmission^18^.

The main focus of this work is the analysis of an apparently simple interpretation problem: in a POD clinical trial, how does one distinguish between a vaccine that prevents fast progression upon infection, or reinfection, from a vaccine that delays it? Two main types of trial designs are analyzed in this work: trials such as the one conducted for the MVA85A vaccine^9^, conducted in cohorts of naive individuals, and trials such as the study for the M72/AS01E vaccine^11^, which involved the recruitment of already- sensitized subjects. In both cases, we formally describe the issue and characterize its negative impact on our ability to produce unbiased impact evaluations for vaccines. Finally, we propose an additional set of analyses that allow us to distinguish among the possible vaccine mechanisms at play and discuss their range of applicability under different epidemiological scenarios, age of participants, trial dimensions and designs.

## Results

### Mapping prevention readouts onto multiple vaccine mechanisms

In an elementary version of *M.tb*. transmission models (Fig. 1a), susceptible individuals (S) are defined by their absence of immunoreactivity to TB—typically showing negative results to an Interferon-gamma-release assay^19^ (IGRA)—and get infected at a rate $\beta$. Upon infection, they split between two classes of infected individuals: F—fast progression to disease—with probability $p$, or L, associated with LTBI, with the remaining probability $1-p$. Individuals in groups $F$ and $L$ differ in their risk to develop active TB per unit time. While fast progressors develop the disease (D) at a rate $r$ associated with typical transition times lower than 2 years^14^, LTBI individuals can remain so for decades^20^, only eventually falling sick, at a rate *r*_L_ ≪ *r*. Furthermore, latently infected individuals can get re-infected, after which, a fraction of them will progress rapidly to disease too. This event occurs at a rate that is proportional to the product of the basal infection rate times the probability of fast progression upon infection ($\beta p$), modulated by a coefficient $q$ that accounts for the protection against fast progression to TB upon reinfection that LTBI confers^21^. For vaccinated subjects, parameters $\beta$, $p$ and $r$ may be reduced to $(1-{\varepsilon }_{\upbeta })\beta$, $(1-{\varepsilon }_{{\rm{p}}})p$ and $(1-{\varepsilon }_{{\rm{r}}})r$, respectively, as a consequence of the action of the vaccine (in all three cases $\varepsilon\, <\, 1$). Typically, trial duration is too short, and cohort size too small, to observe protective effects related to the rate of progression to disease from LTBI (see Supplementary Methods, module I, and Supplementary Fig. 1).

When a clinical trial is conducted in cohorts of susceptible individuals (IGRA negative), the entire dynamical process represented in Fig. 1a can be observed within the context of the study. Concerning the infection end point, it is usually addressed by IGRA conversion, while disease is defined upon standard TB diagnosis criteria^9^. The classical approach to interpret the results of these studies consists of analyzing the times elapsed until requirements of infection and disease end points are verified (Fig. 1b, c), to infer two independent efficacy parameters by using survival analysis: efficacy against infection ${\text{VE}}_{\text{inf}}$ and against disease ${\text{VE}}_{\text{dis}}$ (i.e. POI and POD^12^). However, according to the transmission model in Fig. 1a, these two vaccine efficacy observations can arise from at least three independent mechanisms: reduction of susceptibility to infection (via ${\varepsilon }_{\upbeta }\,> \, 0$), reduction of the probability of fast progression (${\varepsilon }_{{\rm{p}}}\,> \, 0$) and reduction of the rate of fast progression to disease (${\varepsilon }_{{\rm{r}}}\,> \, 0$).

All that said, the nature of the question under analysis turns evident: how to estimate three independent vaccine mechanisms $({\varepsilon }_{\upbeta },{\varepsilon }_{{\rm{p}}},{\varepsilon }_{{\rm{r}}})$ from only two measurements of vaccine efficacy $({\text{VE}}_{\text{inf}},{\text{VE}}_{\text{dis}})$? Regarding POI, we can match the efficacy measured to a reduction in the probability of getting infected upon contact with an infectious individual: ${\text{VE}}_{\text{inf}}\equiv {\varepsilon }_{\upbeta }$. Instead, vaccine’s POD is more complex, and a single readout of ${\text{VE}}_{\text{dis}}$ is compatible with different combinations of effects on fast progression probabilities and transition rates to disease (see Suppplementary Methods, module II and Supplementary Fig. 2). This can be demonstrated mathematically, by deriving a relation ${\text{VE}}_{\text{dis}}=f({\varepsilon }_{{\rm{p}}},{\varepsilon }_{{\rm{r}}},{\varepsilon }_{\upbeta })$ that bounds the parameters (Fig. 1d, case where ${\text{VE}}_{\text{inf}}={\varepsilon }_{\upbeta }=0$, see Supplementary Methods, module II). Importantly, this issue is an unavoidable consequence of incubation periods of fast progression to TB being of the same order of the maximum follow-up periods affordable in this type of trials. This makes it possible to confound an eventual delay in incubation (i.e. ${\varepsilon }_{{\rm{r}}}$) with genuine vaccine-mediated prevention of fast progression to TB (i.e. ${\varepsilon }_{{\rm{p}}}$). In this sense, this is not an artefact of the modelling architecture chosen, and the same ambiguity can be easily parameterized choosing other possible architectures, as long as these include a description of the time of incubation of fast progression to TB^17^.

Once the problem is identified, we interrogate whether vaccines acting through different combinations of (${\varepsilon }_{{\rm{p}}},{\varepsilon }_{{\rm{r}}}$) that are compatible with a common value of ${\text{VE}}_{\text{dis}}$ also produce equivalent impacts when applied on large populations. To answer this question, we capitalized on a model designed to describe *M.tb*. transmission in trans-national settings^16^ (Supplementary Methods, module III, and Supplementary Fig. 3). By using this model, we simulated the introduction of different types of vaccines at the end of 2025, in a high-burden country such as Ethiopia, and estimated their impact, measured as the total number of TB cases prevented until 2050, upon a future immunization campaign targeting newborns, assuming, in an ideal scenario, $100 \%$ vaccine coverage and long-lasting vaccine effects (Fig. 1e). For this particular case, a vaccine preventing fast progression to disease (via ${\varepsilon }_{{\rm{p}}}$) is expected to prevent as many as 256,000 more TB cases (95% CI: 104–466 × 10^3^) than a vaccine based on delaying it (via ${\varepsilon }_{{\rm{r}}}$), even if the values of these parameters in either case (${\varepsilon }_{{\rm{p}}}=0.5$ vs ${\varepsilon }_{{\rm{r}}}=0.74$) are compatible with the same efficacy readout ${\text{VE}}_{\text{dis}}=50 \%$ obtained from a 4-year trial. This amounts to a relative difference of 104% (95% CI: 44–180%) with respect to the least favourable case. Such deviation is also significant for more realistic duration of vaccine protection effects (120% (95% CI: 50–219%)) for $1 \%$ immunity waning per year, and 176% (95% CI: 72–319%) for $5 \%$, see Supplementary Methods, module III and Supplementary Fig. 4).

### Gauging vaccine mechanisms from trial data (naive cohorts)

Next, we introduce an analytical approach to estimate independently the different mechanistic contributions to vaccine POD, namely ${\varepsilon }_{{\rm{r}}}$ and ${\varepsilon }_{{\rm{p}}}$. While ${\varepsilon }_{\upbeta }$ is directly equivalent to the POI readout (${\varepsilon }_{\upbeta }\equiv {\text{VE}}_{\text{inf}}$), and can thus be estimated through Cox regression, ${\varepsilon }_{{\rm{r}}}$ and ${\varepsilon }_{{\rm{p}}}$ cannot, since multiple combinations of them are compatible with a single-efficacy readout (Fig. 1d).

**Fig. 2:**
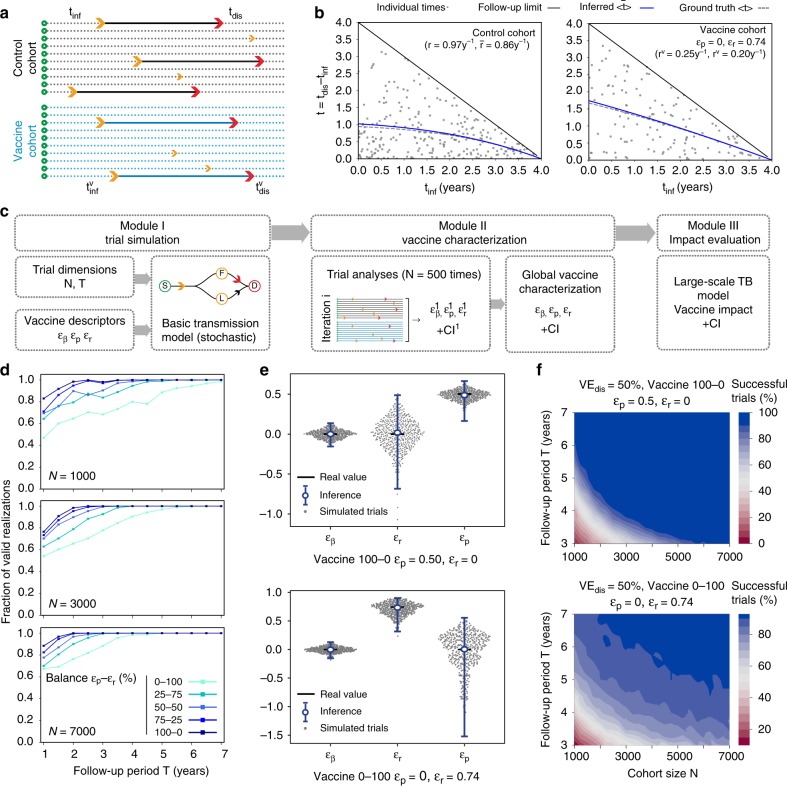
Methods for characterizing vaccine mechanisms from the analysis of clinical trials conducted on naive cohorts. **a** Inference of ${\varepsilon }_{{\rm{r}}}$. From the distribution of times from infection (orange) to disease (red), we obtain the rates of fast progression to TB in each cohort: either ${r}^{{\rm{c}}}=r$ (control), or ${r}^{{\rm{v}}}=r(1-{\varepsilon }_{{\rm{r}}})$ (vaccine). **b** Transition times of control (left) and vaccinated cohort (right: vaccine acting through ${\varepsilon }_{{\rm{r}}}$) between IGRA conversion and disease onset. By using likelihood maximization, we infer within-cohort transmission rates to disease ${r}^{{\rm{c}}}$ an ${r}^{{\rm{v}}}$, which are associated with expected values for the transition times (blue, continuous lines) that closely resemble the a priori-known analytical predictions (dashed lines). From these estimates, ${\varepsilon }_{{\rm{r}}}$ is estimated as $1-{r}^{{\rm{v}}}/{r}^{{\rm{c}}}$. **c** Schematic representation of the computational pipeline used in this work for the analysis of clinical trials conducted on IGRA-negative cohorts, structured in three modules. Module I: trial simulation: From a given vaccine (${\varepsilon }_{\upbeta },{\varepsilon }_{{\rm{r}}},{\varepsilon }_{{\rm{p}}}$) and trial dimensions ($N,T$) we simulate 500 equivalent trials. Module II: vaccine characterization: then, we analyze the outcomes of the simulated trials to estimate ${\varepsilon }_{\upbeta }$, ${\varepsilon }_{{\rm{r}}}$ and ${\varepsilon }_{{\rm{p}}}$. Module III: impact evaluation. We use the comprehensive transmission model developed in ref. ^16^ to evaluate the impact associated with the characterized vaccines. **d** Fraction of valid realizations of a trial yielding epidemiologically plausible vaccine parameterizations, (excluding failed attempts). **e** Vaccine characterization of ${\varepsilon }_{\upbeta },{\varepsilon }_{{\rm{r}}},{\varepsilon }_{{\rm{p}}}$. Error bars represent the 95% confidence interval. **f** Estimated probability of obtaining a trial result, leading to a successful characterization of ${\varepsilon }_{{\rm{p}}}$ (up) or ${\varepsilon }_{{\rm{r}}}$ (bottom) (CI not crossing 0 at a 95% confidence level for the parameter whose ground-truth value is non-zero).

To solve this issue, in addition to the custom estimation of the efficacy against infection ${\text{VE}}_{\text{inf}}\equiv {\varepsilon }_{\upbeta }$ and disease ${\text{VE}}_{\text{dis}}$, we add a third independent statistical analysis to estimate ${\varepsilon }_{{\rm{r}}}$ from the comparison of the transition times between end-point infection and end-point disease across cohorts (Fig. 2a). To do so, we assume that all TB cases observed in a trial correspond exclusively to fast progressors (see Supplementary Methods, module I and Supplementary Fig. 1). This allows us to derive an analytical expression for the expected distribution of transition times observed between IGRA conversion and TB diagnosis $t={t}_{\text{dis}}-{t}_{\text{inf}}$, conditioned to the moment IGRA conversion happened. This distribution $t=\Psi ({r}_{\text{cohort}},{t}_{\text{inf}})$ has as its only parameter the transition rate to disease of the cohort under analysis: $r$, or $r(1-{\varepsilon }_{{\rm{r}}})$ for the control and vaccine group, respectively. We infer these parameters from the data in both cohorts, by using a maximum-likelihood approach, and compare them to estimate ${\varepsilon }_{{\rm{r}}}$ (see Fig. 2b, and Methods).

Thanks to this independent estimation of ${\varepsilon }_{{\rm{r}}}$, we can now use the analytical relationship ${\text{VE}}_{\text{dis}}=f({\varepsilon }_{\upbeta },{\varepsilon }_{{\rm{r}}},{\varepsilon }_{{\rm{p}}})$ previously derived (see Fig. 1d), to solve for the only parameter that remains unknown: ${\varepsilon }_{{\rm{p}}}$, as detailed in the Supplementary Methods, module II. As a result, we obtain a full description of the vaccine through the estimations of the three effects $({\varepsilon }_{\upbeta },{\varepsilon }_{{\rm{r}}},{\varepsilon }_{{\rm{p}}})$ that drive both POI and POD.

We then used Monte-Carlo methods to test the performance of our approach, by simulating clinical trials of different dimensions for different vaccines (Fig. 2c)^22^. For a cohort size $N$ and follow-up period $T$, we use as inputs the ground-truth values of the vaccine parameters $({\varepsilon }_{\upbeta },{\varepsilon }_{{\rm{r}}},{\varepsilon }_{{\rm{p}}})$, and simulate the stochastic development of possible realizations of the trial by using an agent-based implementation of the model represented in Fig. 1a. The outcome of such simulation is a set of two vectors of transition times to infection and TB across participants. Since the model is stochastic, we iterate to obtain a set of simulated trials that yield a distribution of most-likely outcomes conditioned both by trial dimensions and vaccine characteristics. For each simulation, we apply our method to characterize the vaccine and evaluate its goodness comparing the results with the a priori-known ground-truth values.

As a first metric to quantify our method’s performance, we need to ensure that the estimates produced lie within epidemiologically meaningful ranges often enough. To ensure that, we define such meaningful intervals for the vaccine-mediated reduction of fast transition rates (${\varepsilon }_{{\rm{r}}}$) and probability of fast progression (${\varepsilon }_{{\rm{p}}}$) by imposing a series of basic requisites (see Methods: a vaccine cannot delay fast progression to disease to make it slower than slow progression, or modify probabilities of fast progression that go beyond the interval $[0,1]$, etc.). Then, we identify the simulations, that due to insufficient statistics, derive into parameter estimates that go beyond those intervals, and label them as failed attempts. In Fig. 2d we represent the fraction of simulations yielding valid inferences of vaccine descriptors, for the vaccines in Fig. 1d.

As for the comparison between the distribution of inferred estimates and ground-truth values, our method succeeds at producing median estimators that closely resemble the ground truth for different vaccines (Fig. 2e: maximum deviation between median estimates and ground-truth values equal to 0.03 s.d.), albeit a vast uncertainty was caused by the low sample size.

Uncertainty may thus compromise the feasibility of our approach, especially if trials are too small, or brief, to ensure sufficient statistics. To shed light on this issue, we simulated trials of different sizes and durations for the two vaccines represented in Fig. 2e, and obtained, in each case, the probability of obtaining a valid simulation yielding an inferred parameter for the driver mechanism that is valid (thus excluding failed trial attempts), and statistically significant (95% CI not crossing zero). As we see in Fig. 2f, a vaccine that reduced the probability of fast progression (${\varepsilon }_{{\rm{p}}}$, up) is easier to characterize than a vaccine that delays it (${\varepsilon }_{{\rm{r}}}$, bottom). For a trial of $N=3000$ and $T=4$ years, the first vaccine will be successfully characterized with $p=0.95$, while for the second vaccine, that probability of success goes down to $p=0.75$.

In addition, we were interested in addressing how robust the performance of our approach is to variation in the basal epidemiological parameters, especially those that are known to vary more significantly across epidemic settings and age strata: the infection rate $\beta$ and the probability of fast progression $p$. To do that, we simulated and analyzed ensembles of 500 trials under alternative scenarios where $\beta$, $p$ or the level of protection against disease of LTBI individuals, $q$, were allowed to vary around their reference values ($\beta =(0.03,0.05,0.069,0.09,0.11)$, $p=(0.1,0.15,0.2,0.3,0.375,0.5)$ $q=(0.1,0.15,0.21,0.3,0.4)$, see Supplementary Fig. 5). Reassuringly, the method performance is similar in all these alternative scenarios compared with what is shown in Fig. 2, yielding parameter estimates that deviate marginally with respect to the ground-truth values (bias lower than $0.06$ s.d. in all scenarios tested there). This includes incidence rates lower than those observed in the reference setup (down to $\beta =0.03$, bias lower than 0.065 s.d. for each of the parameters, in either ${\varepsilon }_{{\rm{r}}}$ or ${\varepsilon }_{{\rm{p}}}$-based vaccines), and lower values of the fast progression probability, comparable to those typically assumed for adolescents and adults ($p=0.15$, bias lower than 0.021 s.d.)^16^.

Finally, while all the results presented in Fig. 2 correspond to a vaccine that provides POD, but not POI (i.e. ${\varepsilon }_{\upbeta }=0$), in Supplementary Fig. 6 we show that for vaccines conferring at the same time significant levels of POI and POD, the methods presented here can be equally applied, even if, in this scenario, the mechanistic variability underlying POD becomes quantitatively less important.

### Impact evaluation of empirically characterized vaccines

In the previous sections, we described our method to estimate the different mechanistic contributions to vaccine POD from the analysis of IGRA-negative trials data. We also illustrated, in Fig. 1e, how the impacts that vaccines leaning on different combinations of these mechanisms compare. However, in that analysis, the uncertainty of impact estimates does not come from vaccines descriptions, which were still considered error-free, but was propagated from the inputs of the transmission model used (Supplementary Methods, module III). Therefore, it remains pending to address what is the role of the additional uncertainty introduced in impact forecasts that is due to our limited resolution when estimating vaccine parameters.

**Fig. 3:**
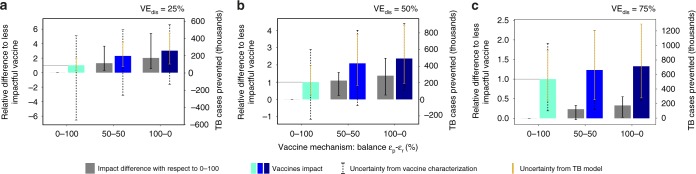
Impact evaluation of empirically characterized vaccines: mechanism effects on expected impact and uncertainty. **a**, **b**, **c** Vaccines characterized from an efficacy readout of ${\text{VE}}_{\text{dis}}=25 \%$, ${\text{VE}}_{\text{dis}}=50 \%$ and ${\text{VE}}_{\text{dis}}=75 \%$, respectively. Blue bars: impact estimates for different vaccines. Two different contributions to the overall impact uncertainty are distinguished: Gold bars: intrinsic contribution coming from general inputs of the transmission model. Black, dashed bars: extra contribution from uncertain vaccine characterizations. Grey bars: differences in impact between each vaccine and the least impactful case of a vaccine acting 100% through ${\varepsilon }_{{\rm{p}}}$ (leftmost, light blue bar in each panel). Error bars represent the 95% confidence interval.

To answer this question, we turn to the model used to estimate vaccine impacts in large-scale settings described before (Fig. 1e), which we now use to estimate how does the uncertainty in vaccine characterization propagate into impact evaluations. To do that, we simulate sets of trials for vaccines of efficacy against disease (${\text{VE}}_{\text{dis}}$) of 25, 50 and $75 \%$, leaning on different combinations of the effects on fast transition rates (${\varepsilon }_{{\rm{r}}}$) and probabilities (${\varepsilon }_{{\rm{p}}}$). Then, we use our inference method to estimate the values of these parameters, and use those estimates, along with their corresponding aggregated uncertainty intervals, to feed the transmission model and estimate vaccine impact. The site and period chosen for vaccine evaluation correspond to Ethiopia for a hypothetical vaccination strategy implemented on newborns between the end of 2025 and 2050. The results of these analyses are shown in Fig. 3. As shown before for the reference case of ${\text{VE}}_{\text{dis}}=50 \%$, for efficacy equal to 25 and 75%, we also observe significant differences in impact when comparing vaccines that depend on the two mechanisms studied (relative difference between a ${\varepsilon }_{{\rm{p}}}$-based vaccine and a ${\varepsilon }_{{\rm{r}}}$ vaccine equal to 201% (95% CI: 52–541%) for ${\text{VE}}_{\text{dis}}=0.25$, and 32% (95% CI: 2.5–55%) for ${\text{VE}}_{\text{dis}}=0.75$, with respect to the vaccine acting through ${\varepsilon }_{{\rm{r}}}$).

Concerning impact uncertainty, vaccine characterization adds to the rest of uncertainty sources of the transmission model, contributing to total impact CIs with a fraction that varies from 3.3 to 85.3%, depending on vaccine efficacy levels and mechanisms. Under our transmission model, this uncertainty prevents us from rejecting the null hypothesis of null impact in more than one-half of the cases explored: those of vaccines based exclusively on ${\varepsilon }_{{\rm{r}}}$ and/or those characterized by low ${\text{VE}}_{\text{dis}}$ ($25 \%$), as well as for the mixed vaccine with ${\text{VE}}_{\text{dis}}=50 \%$. Be it as it may, Fig. 3 highlights again the pertinence of our approach, since the differences between the impacts estimated by vaccines leaning on different combinations of ${\varepsilon }_{{\rm{r}}}$–${\varepsilon }_{{\rm{p}}}$ for the same values of ${\text{VE}}_{\text{dis}}$ (grey bars) are still significant regardless of how uncertain vaccine characterization is.

### Clinical trials conducted on IGRA-positive cohorts

**Fig. 4:**
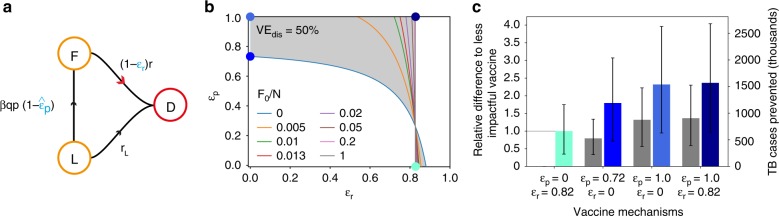
Vaccine characterization from clinical trials conducted on IGRA-positive individuals. **a** Section of the transmission chain that is observed during a trial conducted on cohorts of IGRA-positive individuals. Recruiting IGRA-positive participants turns possible to observe a vaccine-mediated protection against fast progression to TB upon reinfection during the trial (i.e. $\hat{{\varepsilon }_{{\rm{p}}}}$), in addition to a delay in the transition rate to disease (${\varepsilon }_{{\rm{r}}}$). **b** Family of curves that bound ${\text{VE}}_{\text{dis}}$, $\hat{{\varepsilon }_{{\rm{p}}}}$ and ${\varepsilon }_{{\rm{r}}}$ for different levels of the fraction of individuals recruited within the reservoir $F$: $F/(L+F)$. The shaded area represents the whole set of points ($\hat{{\varepsilon }_{{\rm{p}}}}$,${\varepsilon }_{{\rm{r}}}$) that are compatible with a single readout ${\text{VE}}_{\text{dis}}=50 \%$, when the basal epidemiological parameters of the population are the same used in the previous sections. The dots represent four extreme vaccine examples whose impacts are to be estimated later. **c** Different impacts foreseen from the four extreme vaccines highlighted in **b** (blue bars), and differences with respect to the least favourable interpretation (grey bars). Error bars (black bars) represent the 95% confidence interval.

The results presented in previous sections pertain to clinical trials conducted on cohorts of IGRA-negative individuals. However, the results from the candidate M72/AS01E^11^ have shifted the focus to an alternative design, based on the recruitment of IGRA-positive subjects (Fig. 4a). In this case, we could expect to find two different subpopulations of individuals in each of the cohorts. First, a group of subjects who were infected on average a long time ago, and are assumed to be LTBI carriers, were characterized by a low risk of endogenous progression to active TB (slow-latency reservoir, $L$). On the other hand, we will have a second type of participants, who generally were infected more recently and who would be progressing through the subclinical TB spectrum^23^, and are thus at a high risk of progressing to active disease in the next few months (i.e. fast-latency reservoir $F$). Considering that, it is evident that in this case, the same kind of multiple-interpretation issue that we described above is equally pertinent. Now, the vaccine might be delaying the progression to disease of the second group (through ${\varepsilon }_{{\rm{r}}}$), or it might protect the individuals of the first group against disease progression after a secondary infection event registered during the trial (an effect that we parameterize as ${\hat{\varepsilon }}_{p}$, which would relate to what we observed in the previous sections through the relation ($1-{\hat{\varepsilon }}_{{\rm{p}}}$)$\equiv$($1-{\varepsilon }_{\upbeta }$)($1-{\varepsilon }_{{\rm{p}}}$). As for the eventual effect of a vaccine on ${r}_{{\rm{L}}}$, this could possibly be observed only if, at the same time, we have an arguably prohibitive cohort size and/or trial duration, as well as a negligible contribution of fast progressors or reinfections (meaning, being in a low-burden setting). Since these conditions are not met in the type of studies that the community is currently engaged in refs. ^9,11^, the observation of these effects in the trials here analyzed would be extremely unlikely, and therefore, we decided not to introduce it in our models.

Despite such simplifying assumption, now it is harder to distinguish the different dynamical mechanisms that could be at play from analytical means alone. This is because of two main reasons. First, we can derive, similar to the IGRA-negative case, an analytical relationship between a readout of efficacy against disease ${\text{VE}}_{\text{dis}}$, and the vaccine effects on fast progression probabilities and rates: ${\hat{\varepsilon }}_{{\rm{p}}}$−${\varepsilon }_{{\rm{r}}}$ (see Supplementary Methods, module II). However, the relationship has now an unknown free parameter—the relative weight of individuals in the two subpopulations $F$ and $L$—that turns the curve of values ${\hat{\varepsilon }}_{{\rm{p}}}$−${\varepsilon }_{{\rm{r}}}$ compatible with a given efficacy readout of ${\text{VE}}_{\text{dis}}$, and an estimation of ${\varepsilon }_{\upbeta }$, into the envelope of a whole family of curves (shaded area in Fig. 4b). This exacerbates, by construction, the multiplicity of different combinations of vaccine mechanisms that could underlie the readout of a trial. The second reason is that, even if we knew how many of the individuals begin the trial in the $F$ vs $L$ reservoirs, we would not have enough information to estimate independently the eventual vaccine-mediated delay of incubation periods of fast progressors ${\varepsilon }_{{\rm{r}}}$, for the times at which these were infected would still be unknown.

As a consequence, the interpretation of the outcomes of a trial, such as the one of M72/AS01E^11^, is hindered by the very study design, and as a consequence, the uncertainty of any vaccine impact evaluation that does not obviate the possibility of observing different vaccine mechanisms of action gets compromised. As shown in Fig. 4c this translates into a wide variety of possible impacts (maximum impact is $136 \%$ higher than the minimum one (95% CI: 58–229$\%$)), all of them associated with vaccines compatible with a single readout of ${\text{VE}}_{\text{dis}}=50 \%$, that adds up to the uncertainty that is intrinsic to the production of model-based forecasts themselves.

The results in Fig. 4 correspond to a vaccine of ${\text{VE}}_{\text{dis}}=50 \%$, analyzed on a trial conducted on a population affected by the same epidemiological parameters used in Figs. 1–3, with the exception of the probability of fast progression upon infection, that is fixed at $p=0.15$ to capture the situation of adolescents/adults, instead of newborns^16^. This is motivated by the fact that recruitment of IGRA-positive individuals is easier in older individuals than in infants, and as such, this type of design is more commonly considered within the context of studies conducted on adolescents and/or adults (see, e.g. ref. ^11^). The impacts evaluated in Fig. 4c correspond to a vaccine implemented on adolescents too (applied on 15-year-old individuals), showing that the differential effects of vaccine mechanisms on impact estimates also appear beyond the context of vaccines applied on newborns.

## Discussion

As we discussed above, in clinical trials of TB vaccines conferring POD, vaccine protection can be attributed to several dynamical mechanisms. More specifically, a vaccine can provide protection by either slowing down fast progression to disease, or by preventing it, and these mechanisms cannot be disentangled by classical survival analysis alone. This makes trial readouts hard to reconcile with transmission model architectures, which constitutes an unexpectedly relevant issue because, as we show, vaccines that differ in the mechanisms through which POD takes place are expected to cause significantly different impacts, even when they appear as equally effective in the context of a clinical trial. In this regard, our results indicate that prevention of fast progression to TB upon infection should be recognized, unlike the delay of incubation periods, as a preferred product characteristic for TB vaccines^24^. Vaccines that are based on a delay of incubation are comparatively less impactful and harder to characterize successfully than their counterparts. This observation is equally valid regardless of the target age group, for it is robust under a series of alternative epidemiological assumptions, including values of the basal parameters characteristic of both infants and adults.

The problem of identifying the vaccine mechanism at play is harder to tackle depending on the type of trial design. For trials conducted on IGRA-negative cohorts, it is possible to identify the vaccine mechanisms at play, which helps to reduce bias in vaccine evaluations. Instead, a trial design based on the recruitment of IGRA-positive subjects presents intrinsic limitations that prevent the usage of statistical techniques to distinguish between the mechanisms underlying vaccine protection. In this case, there are two key pieces of information that remain hidden to the modeller: the fraction of recruited participants who are on their way to progression to the active disease at the beginning of the trial, and the times when they were first infected. Without access to these data, the POD readouts obtained from these designs are harder to interpret in terms of transmission models, and impact forecasts derived from them turn extremely uncertain, even more so than these customarily are. It is worth highlighting that this issue  has nothing to do with an additional, and obvious limitation of IGRA-positive designs, namely, that they do not allow estimating possible POI effects. Instead, the problem here described implies that in the absence of further evidence, it is impossible to estimate how impactful the POD effects characterized in one such trial might be in a large-scale setting.

Our conclusions are not exempt of other limitations. First, the possible vaccine mechanisms of action analyzed here are not the only possible. In principle, a vaccine can disrupt the dynamics of the natural history of the disease at any point (see e.g. ref. ^25^), and yet these effects would be virtually impossible to observe in trials within phases 2b/3 such as the ones here discussed. Furthermore, it is important to highlight that model-based impact evaluation of vaccines is always a daunting task, especially in TB. The importance of aspects such as the uneven quality of the empirical evidence behind the many parameters these models rely on, or the assumption that all IGRA-positive readouts can be interpreted as real latent infection cases cannot be overstated^14^. Also, heterogeneities in clinical outcomes due to either host, pathogen or environmental variability impose an additional layer of complexity that goes beyond the phenomena discussed here, whose interaction with vaccine function needs to be assessed too. Concerning the impact estimates that we provide in this study, they have been obtained from vaccine assumptions that are to a great extent an idealization (i.e. 100% coverage levels, long-lasting duration of protection and immediate acquisition of immunity upon vaccination, see Methods, and Supplementary Methods (module III) for details). However, the differences between the cases associated with different mechanisms, which is the main result here presented, are robust under different vaccine scenarios, such as different basal efficacy levels, different ages of the target populations, different levels of protection waning and combinations of POI/POD effects (see Supplementary Methods (module III) and Supplementary Fig. 6).

Concerning our ability to distinguish the vaccine mechanisms at play in the case of IGRA-negative designs, there are two additional limitations to highlight. First, similar to the current methodology, our approach can only be of use if sufficient statistics is available. This means high levels of basal TB incidence, and big enough trial dimensions—time and cohort size—as previously discussed. Finally, an additional limitation concerns the maximum duration of trials for which our methods, in their original form here presented, would still be sensible. This limitation arises because of two different reasons. First, our ability to estimate ${\varepsilon }_{{\rm{r}}}$ relies on the assumption that all individuals that develop active TB during the trial are fast progressors. This assumption is less accurate, and introduces bias, as the follow-up period increases, even if it is still approximately valid until $T\simeq 10y$ (see Supplementary Methods, module I). Second, both infection and disease risks in tuberculosis are known to be strongly age-dependent^16^. This would discourage the usage of a single set of basal epidemiological parameters (mainly $\beta$ and $p$) to describe the behaviour of a cohort, during the entire duration of a study, if this is too long. This would not impose a conceptual hindrance to the applicability of our approach, since the methods here described could be granted with age structure in order to estimate vaccine effects conditioned to the variation of basal epidemiological parameters with age and how vaccine effects change with time since vaccination, provided that enough statistics are available.

Our ultimate goal is to narrow the gap between trial-derived efficacy estimations and model-based impact evaluations. We demonstrated that at least for some particular trials’ architectures, the combination of Monte-Carlo methods and compartmental models constitutes a powerful resource that allows us to make substantial progress in that direction, and to give advice to trial designers about the differential advantages of different possible trial dimensions and designs, beyond other practical implications that are already profusely discussed in the field. As demonstrated in this work, it is necessary to reconcile the interpretation of trial results with the formulation of the mathematical models used to evaluate vaccine impact. This is key in order to reduce uncertainty in impact evaluations, improve the evaluation of candidate vaccines and reduce risk in the decision-making processes of funding agencies and public health authorities. We foresee that this notion will also be relevant for the design and analysis of future, possibly different phase 2b/3 trials of other vaccines.

## Methods

### Module I: in silico clinical trial simulations

To simulate trials on IGRA-negative cohorts, we calibrate the baseline parameters of the transmission model in Fig. 1a to reflect the current epidemic situation in a reference setting. For that, we chose the target cohort of newborns living in Worcester, South Africa, where the MVA85A study took place from 2009 to 2012^9^. The transition rate from LTBI to disease is assumed to be ${r}_{{\rm{L}}}=7.5\times 1{0}^{-4}\,{{\mathrm{y}}}^{-1}$, in accordance with previous bibliographical estimations^15^. According to ref. ^21^, we consider that LTBI individuals have a $79 \%$ less risk of progressing to TB upon reinfection, which is captured by the parameter $q=0.21$. Finally, the probability of fast progression has been fixed to $p=0.375$ that is compatible with previous observations about the high probability of developing fast progression during the first few months of life^26^. Once those parameters are fixed, the baseline transmission rate $\beta$ and the transition rate from fast latency to disease $r$ were estimated to be $\beta =0.069\,{{\mathrm{y}}}^{-1}$ and $r=0.97\,{{\mathrm{y}}}^{-1}$ to reproduce the proportion of infections and TB cases observed in the control cohort of the MVA85A trial (12.8% and 2.3% after 2 years, respectively). Even though these parameters are representative of epidemiological risks of newborns, we also explored alternative scenarios, including parameterizations compatible with other age groups, as detailed in Supplementary Fig. 5.

Next, we arbitrarily define a vaccine by providing the triad of vaccine efficacies $({\varepsilon }_{\upbeta },{\varepsilon }_{{\rm{r}}},{\varepsilon }_{{\rm{p}}})$, describing its effects on the infection rate, the transition rate to disease and the probability of fast progression, respectively. While a value of, for example, ${\varepsilon }_{\upbeta }=0$ means no protection against infection, ${\varepsilon }_{\upbeta }=1$ means total protection.

Once all the dynamical parameters governing *M.tb*. transmission dynamics in both cohorts are set up, we use an agent-based model to describe the evolution of $N$ individuals per cohort during a follow-up period of $T$. Here, we apply Monte-Carlo methods to simulate, individually, the fates that each participant in one such trial might meet according to the different probabilistic risks per unit time of getting infected, eventually re-infected and/or progressing to disease through either the fast or slow routes, as described in detail in the Supplementary Methods, module I. After a trial is simulated, the times when each individual enters into the disease, and/or infection end points are registered, and discretized to a time step of 3 months to reproduce the temporal resolution between consecutive analyses (IGRA for infection and/or TB diagnosis tests for active TB) that one would observe in an actual trial such as the MVA85A study^9^.

### Module II: data analysis of trial outcomes

In practice, the efficacy readouts of POI and POD (${\text{VE}}_{\text{inf}}$ and ${\text{VE}}_{\text{dis}}$) are estimated by using survival analysis (Cox regression^27^). In the case of vaccine POI, the readout of ${\text{VE}}_{\text{inf}}$ obtained this way can be directly associated with a reduction of the risk of infection upon contact with an infectious subject, that is, ${\text{VE}}_{\text{inf}}\equiv {\varepsilon }_{\upbeta }$. However, ${\text{VE}}_{\text{inf}}$ can only be estimated from a trial based on IGRA-negative subjects^9^, but not IGRA-positive^11^.

Vaccine-mediated POD can be estimated from trials recruiting IGRA-negative or IGRA-positive individuals, though. However, the existence of different mechanisms compatible with a single readout of ${\text{VE}}_{\text{dis}}$ poses a series of conceptual challenges to its estimation through classical survival analysis that supports the adoption of the more elementary approximation ${\text{VE}}_{\text{dis}}=1-\rho$, where $\rho$ is the fraction of the total number of diseased individuals observed in both cohorts at the end of the trials: $\rho ={D}_{{\rm{v}}}(T)/{D}_{{\rm{c}}}(T)$. This choice is justified by a series of observations. On the one hand, it permits the derivation of an analytic relation between ${\text{VE}}_{\text{dis}}$ and the mechanistic parameters $({\varepsilon }_{\upbeta },{\varepsilon }_{{\rm{r}}},{\varepsilon }_{{\rm{p}}})$, which is key to our approach. On the other hand, it produces estimates for ${\text{VE}}_{\text{dis}}$ that only deviate residually from the readouts obtained from survival analyses (relative deviation lower than $8 \%$, see Supplementary Methods, module II). Finally, using survival analysis to determine ${\text{VE}}_{\text{dis}}$ is problematic, since at least for some of the possible vaccine mechanisms, the hypothesis of proportional risks, which is the major conceptual requisite for Cox regression to be applied safely, is not respected. These issues are discussed in detail in the Supplementary Methods, module II.

Furthermore, in trials conducted on IGRA-negative cohorts, ${\varepsilon }_{{\rm{r}}}$ can be estimated from a truncated fit of uncensored sub-cohorts’ transition rates. In this case, a vast majority of all TB cases can be expected to correspond to fast progression after the first infection event (see Supplementary Methods, module I). Furthermore, if we assume that transition from active disease upon infection is a Poisson process—as it is customarily assumed in the TB modelling literature^15,28,29^—the theoretical probability distribution function (PDF) of the time $t={t}_{\text{dis}}-{t}_{\text{inf}}$ between infection and disease in the control cohort corresponds to an exponential curve $f(t| r)=r{e}^{-rt}$, from which the average transition time $\langle t\rangle =1/r$ and its associated variance ${\sigma }_{t}^2=\langle {t}^{2}\rangle -{\langle t\rangle }^{2}=1/{r}^{2}$ can be easily obtained by integrating the moments of the PDF.

However, in a clinical trial, the period of measure cannot be arbitrarily extended, which implies that the maximum transition time that can be observed for a subject who was initially infected at ${t}_{\text{inf}}$ is truncated at ${t}_{{\mathrm{max}}}=T-{t}_{\text{inf}}$, where $T$ stands for the follow-up period of the trial. This situation implies that the integrals needed to obtain the expected value of the transition time must be truncated as well, which ultimately makes $\langle t\rangle$ to depend itself on ${t}_{\text{inf}}$:

1 $$\langle t\rangle ({t}_{\text{inf}})=\frac{{\int }_{0}^{T-{t}_{\text{inf}}}tf(t| r)dt}{{\int }_{0}^{T-{t}_{\text{inf}}}f(t| r)dt}=\frac{1}{r}-\frac{{e}^{-r(T-{t}_{\text{inf}})}\left(T-{t}_{\text{inf}}\right)}{1-{e}^{-r(T-{t}_{\text{inf}})}}$$

Similarly, by truncating the integrals of the second moment of the distribution, we can obtain its dependence with time at infection, $\langle {t}^{2}\rangle ({t}_{\text{inf}})$, and ultimately derive the corresponding expression for the variance of observed transition times as a function of ${t}_{\text{inf}}$:

2 $$\begin{array}{lll}{\sigma }_{t}^{2}({t}_{\text{inf}})&=&\displaystyle\frac{-{e}^{-r(T-{t}_{\text{inf}})}\left(1+{(r(T-{t}_{\text{inf}})+1)}^{2}\right)+2\left(1+r(T-{t}_{\text{inf}}){e}^{-r(T-{t}_{\text{inf}})}\right)}{{r}^{2}\left(1-{e}^{-r(T-{t}_{\text{inf}})}\right)}\\ &&\displaystyle-\frac{1}{{r}^{2}}-\frac{{(T-{t}_{\text{inf}})}^{2}{e}^{-2r(T-{t}_{\text{inf}})}}{{\left(1-{e}^{-r(T-{t}_{\text{inf}})}\right)}^{2}}\hfill\end{array}$$

Equations (1) and (2) describe how observed transition times from infection to disease and their variance are expected to be biased towards lower values as the infections occur later during the trial. This is simply because the later the infection takes place, the less time available to observe a transition to disease is left. These expressions allow us to isolate the effect of that bias, and to infer, using only data from individuals developing active TB during the trial, the transition rate $r$ within the control cohort, using a Maximum Likelihood approach (R package bbmle^30^) along with its confidence intervals (95% reported). Then, the exercise is repeated in the vaccine cohort, whose transition rate ${r}^{{\rm{v}}}$, in terms of our transmission model would be expressed as the product $r(1-{\varepsilon }_{{\rm{r}}})$, which yields the following expression for the vaccine effect on the fast progression rate ${\varepsilon }_{{\rm{r}}}$:

3 $${\varepsilon }_{{\rm{r}}}=1-\frac{{r}^{{\rm{v}}}}{r}$$

Finally, we obtain an estimation of the CI for ${\varepsilon }_{{\rm{r}}}$ by propagating the independent uncertainties of ${r}^{{\rm{v}}}$ and $r$.

Our ability to estimate the vaccine-mediated effects on the incubation rates that are captured by ${\varepsilon }_{{\rm{r}}}$ depends, by construction, on being able to observe those times in the context of a trial, implying registering the moment when individuals undergo IGRA conversion, and then, fall sick. Once again, this obviously implies that observing this effect is only possible if we recruit IGRA-negative individuals. In a trial conducted on already-infected subjects, the eventual effects that a vaccine might have on incubation rates could never be isolated.

Once ${\varepsilon }_{{\rm{r}}}$ is obtained from the method described above, the next step consists of inferring the last unknown vaccine mechanism ${\varepsilon }_{{\rm{p}}}$. In a trial conducted on IGRA-negative subjects, the effect of the vaccine on the infection rate is captured by ${\varepsilon }_{\upbeta }\equiv {\text{VE}}_{\text{inf}}$, and as such, can be inferred by using Cox regression (R package OIsurv^27^). Furthermore, ${\varepsilon }_{{\rm{r}}}$ has been independently estimated by analyzing times of progression from infection to disease, as described above. In order to infer the third vaccine effect—reduction of fast progression probability ${\varepsilon }_{{\rm{p}}}$—we seek for an analytical relationship ${\text{VE}}_{\text{dis}}=f({\varepsilon }_{\upbeta }\equiv {\text{VE}}_{\text{inf}},{\varepsilon }_{{\rm{p}}},{\varepsilon }_{{\rm{r}}})$ that will allow us to solve for ${\varepsilon }_{{\rm{p}}}$ once the other parameters, including ${\text{VE}}_{\text{dis}}=1-\rho$, have already been estimated.

We can obtain this relationship, represented in Fig. 1d, by deriving analytical expressions for the expected time evolution of the numbers of susceptible, infected and diseased individuals in a trial. To that end, we now use a deterministic compartmental model based on ordinary differential equations, which is solved as follows. If we call $S$, $F$, $L$ and $D$ the number of individuals in each subpopulation, these evolve in time according to the following coupled differential equations:

4 $$\frac{dS(t)}{dt}=-(1-{\varepsilon }_{\upbeta })\beta S(t)$$

5 $$\frac{dF(t)}{dt}=(1-{\varepsilon }_{\upbeta })\beta (1-{\varepsilon }_{{\rm{p}}})pS(t)-(1-{\varepsilon }_{{\rm{r}}})rF(t)+(1-{\varepsilon }_{\upbeta })\beta (1-{\varepsilon }_{{\rm{p}}})pqL(t)$$

6 $$\frac{dL(t)}{dt}=(1-{\varepsilon }_{\upbeta })\beta (1-(1-{\varepsilon }_{{\rm{p}}})p)S(t)-{r}_{{\rm{L}}}L(t)-(1-{\varepsilon }_{\upbeta })\beta (1-{\varepsilon }_{{\rm{p}}})pqL(t)$$

7 $$\frac{dD(t)}{dt}=(1-{\varepsilon }_{{\rm{r}}})rF(t)+{r}_{{\rm{L}}}L(t)$$

where the three vaccine descriptors $({\varepsilon }_{\upbeta },{\varepsilon }_{{\rm{p}}},{\varepsilon }_{{\rm{r}}})$ are absent (i.e. set to zero) in the control cohort. In this model, we implicitly assume that the individuals in the cohorts correspond to a small fraction of the total population (of the country, area, etc. being modelled), and thus, their contribution to overall transmission once they are sick can be neglected. By integrating this model analytically and independently in each cohort (see Supplementary Methods, module II), we can define the disease ratio $\rho \equiv {D}_{{\rm{v}}}(t)/{D}_{{\rm{c}}}(t)$, and obtain an analytical expression for it that depends on the observation time $t$, and the vaccine descriptors $({\varepsilon }_{\upbeta },{\varepsilon }_{{\rm{p}}},{\varepsilon }_{{\rm{r}}})$:

8 $$\rho =\rho (t,{\varepsilon }_{\upbeta },{\varepsilon }_{{\rm{p}}},{\varepsilon }_{{\rm{r}}})$$

Then, we evaluate $\rho (t=T,{\varepsilon }_{\upbeta },{\varepsilon }_{{\rm{p}}},{\varepsilon }_{{\rm{r}}})$ at the end of the trial, along with its uncertainty, which is propagated assuming that both ${D}_{{\rm{v}}}$ and ${D}_{{\rm{c}}}$ come from two independent binomial distributions (total number of tests equal to cohort size). Finally, by using the independent estimators of ${\varepsilon }_{\upbeta }$ and ${\varepsilon }_{{\rm{r}}}$ as well as their uncertainty estimates, obtained as detailed above, we get our final estimate of ${\varepsilon }_{{\rm{p}}}$ and propagate its corresponding confidence interval. As discussed before, the disease ratio $\rho$ defines by itself our estimate of ${\text{VE}}_{\text{dis}}$, since ${\text{VE}}_{\text{dis}}=1-\rho$. This is the reason why the functional relationship $\rho (t=T,{\varepsilon }_{\upbeta },{\varepsilon }_{{\rm{p}}},{\varepsilon }_{{\rm{r}}})$ can also be expressed as ${\text{VE}}_{\text{dis}}({\varepsilon }_{\upbeta },{\varepsilon }_{{\rm{p}}},{\varepsilon }_{{\rm{r}}})$, or in the specific case where ${\varepsilon }_{\upbeta }$ is assumed to be known (after survival analysis) as ${\text{VE}}_{\text{dis}}({\varepsilon }_{{\rm{p}}},{\varepsilon }_{{\rm{r}}})$, as in Fig. 2a, for example.

As noted previously, it is expected that the intrinsic efficacies of the vaccine are comprised between 0 and 1, where 1 would mean total efficacy and 0 no effect at all. However, it is possible for a vaccine to have a negative effect. In the case of efficacies affecting rates (i.e. ${\varepsilon }_{\upbeta }$ and ${\varepsilon }_{{\rm{r}}}$) there is no formal lower limit and a rate equal to $\infty$ (associated with $\varepsilon =-\infty$) would mean an instantaneous process, although a conservative enough limit of −300 is implemented to avoid numerical instabilities. On the contrary, ${\varepsilon }_{{\rm{p}}}$ works as a modifier of a probability, which implies that $(1-{\varepsilon }_{{\rm{p}}})p$ has to be comprised between 0 and 1, by introducing a lower limit for ${\varepsilon }_{{\rm{p}}}$, i.e. ${\varepsilon }_{{\mathrm{p}},\text{min}}=1-\frac{1}{p}$. Furthermore, the existence of such lower bound in ${\varepsilon }_{{\rm{p}}}$ generates in turn an upper bound for ${\varepsilon }_{{\rm{r}}}$, since these two parameters are bound (see Supplementary Methods, module II) through the relationship ${\text{VE}}_{\text{dis}}({\varepsilon }_{\upbeta },{\varepsilon }_{{\rm{p}}},{\varepsilon }_{{\rm{r}}})$. Notwithstanding this, the inference of ${\varepsilon }_{{\rm{r}}}$ is agnostic to the value of $\rho$ or ${\varepsilon }_{{\rm{p}}}$, and as a consequence, for poor statistical settings—most often in the case of vaccines delaying fast progression—some individual trial realizations lead to vaccine descriptor estimates that lie beyond these epidemiologically meaningful intervals for parameters ${\varepsilon }_{{\rm{p}}}$ and ${\varepsilon }_{{\rm{r}}}$.

In order to obtain global estimates and confidence intervals for vaccine descriptors, we follow a three-step approach. First, we generate a set of 500 synthetic clinical trials for each vaccine analyzed. Second, for each of these simulated trials, we infer the values of the vaccine descriptors ${\varepsilon }_{\upbeta },{\varepsilon }_{{\rm{p}}}$ and ${\varepsilon }_{{\rm{r}}}$ along with their confidence intervals: that of ${\varepsilon }_{\upbeta }$ from Cox regression, that of ${\varepsilon }_{{\rm{r}}}$, propagated from the maximum-likelihood estimates of ${r}^{{\rm{c}}}$ and ${r}^{{\rm{v}}}$ and finally that of ${\varepsilon }_{{\rm{p}}}$ propagated from the other two, and from the CI of the disease ratio $\rho$, as explained in more detail in the Supplementary Methods, module II. Finally, we assume that the true values of these parameters come from an unweighted mixture of normal distributions, each of which is associated with the log transform of one minus the outcome of each simulated trial. The final value and CI of each of the three vaccine descriptors are associated with the median and 95% CI of such distribution mixture, back in the linear scale. Through this approach, we get a global estimation of the accuracy and precision of our method as a function on the predefined vaccine’s characteristics and trial dimensions, which we have introduced in the TB-spreading model for the forecasts of vaccine impacts (and their corresponding confidence intervals).

In the case of trials conducted on IGRA-positive cohorts, the system of equations analogous to eqs. (4)–(7), describes the model depicted in Fig. 4a:

9 $$\frac{dF(t)}{dt}=-(1-{\varepsilon }_{{\rm{r}}})rF(t)+(1-{\hat{\varepsilon }}_{{\rm{p}}})\beta pqL(t)$$

10 $$\frac{dL(t)}{dt}=-{r}_{{\rm{L}}}L(t)-(1-{\hat{\varepsilon }}_{{\rm{p}}})\beta pqL(t)$$

11 $$\frac{dD(t)}{dt}=(1-{\varepsilon }_{{\rm{r}}})rF(t)+{r}_{{\rm{L}}}L(t)$$

Despite their apparent simplicity, these equations hide a very relevant hindrance with respect to the previous case, namely, the fact that, as detailed in the Supplementary Methods, module II, their solution introduces an additional parameter ${F}_{{\mathrm{o}}}$, that represents the initial number of fast progressors who are recruited in the cohorts (even assuming it is the same in both) at the beginning of the trial. This parameter, that in principle cannot be easily determined during trial recruitment, introduces an unknown degree of freedom in the functional relationship between ${\text{VE}}_{\text{dis}}$ and the vaccine parameters, which now would be expressed as ${\text{VE}}_{\text{dis}}=f({F}_{{\mathrm{o}}},{\varepsilon }_{\upbeta },{\hat{\varepsilon }}_{{\mathrm{p}}},{\varepsilon }_{{\rm{r}}})$. As a result, where we had a single curve to capture the relation between ${\varepsilon }_{{\rm{p}}},{\varepsilon }_{{\rm{r}}}$ and ${\text{VE}}_{\text{dis}}$ (for ${\varepsilon }_{\upbeta }=0$, Fig. 1d), we have now the envelope of a parametric family of curves (Fig. 4b).

### Module III: model-based impact evaluations of TB vaccines

Once we have discussed how to characterize a given vaccine from the outcomes of different types of trials, we evaluate and compare the potential impact of these hypothetical vaccines when applied on larger populations. To do so, we take advantage of the detailed *M.tb*. transmission model described in ref. ^16^, developed by the authors as a tool for the description of *M.tb*. transmission in *trans*-national settings characterized by different TB burden levels, different demographic trends and mixing patterns across age groups. Conceptually, this model is a generalization of the increased complexity of the reduced transmission model sketched in Fig. 1a and formalized around the simple system of ordinary differential equations shown through Eqs. (4)–(7). The most important difference between both formulations is that while the elementary transmission model described in this work is suited to capture the time evolution of the fraction of susceptible, infected and sick individuals only within the trials’ cohorts and only during the development of the study, the more complex version developed in ref. ^16^ describes the situation in the entire population, during larger time spans (decades). In our case, the model is calibrated to reproduce TB incidence and mortality rates in Ethiopia in the period 2000–2015. Once the model is calibrated, we use it to produce forecasts until 2050 under two different scenarios: one scenario of no intervention and another one where a vaccine is introduced by the end of 2025. Then, we obtain impact estimates of the different vaccines analyzed in this study as the difference in total TB cases between those two scenarios.

The technical specifications of this detailed age-structured model of *M.tb*. transmission can be found in ref. ^16^ and in the Supplementary Methods (module III). It includes two cohorts of individuals—vaccinated and non-vaccinated—two paths to disease—fast and slow—and six different situations of disease, depending on treatment status (present or absent) and on its aetiology—pulmonary (smear positive/negative) vs non-pulmonary. Regarding treatment results, the model explicitly describes the main outcomes defined by WHO data schemes: treatment completion, default, failure and death, as well as natural recovery. Furthermore, several types of infection events are taken into account, including infection of previously unexposed individuals, exogenous reinfections of previously infected subjects and mother–child transmission. Beyond these considerations that affect the way that pathogen’s transmission is described within each age group, the model contemplates that the parameters governing the dynamics are, as it is usually done in TB modelling, different across age groups. It also contemplates more innovative ingredients, such as heterogeneous contact patterns among age groups that have been adapted from empirical survey studies, and a way to describe the effects of population’s ageing on the transmission dynamics of the pathogen. To do this, the model integrates data from assorted bibliographic sources (see ref. ^16^ for further details), demographic data from the UN population division database^31^ as well as aggregated burden estimates reported for Ethiopia between 2000 and 2015^32^.

For further details regarding the specific values of epidemiological parameters, vaccine descriptions and uncertainty estimates, the reader is referred to the Supplementary Methods, module III.

### Reporting summary

Further information on research design is available in the Nature Research Reporting Summary linked to this article.

## Supplementary information

Supplementary Information

Reporting Summary

## Data Availability

The data underlying the results presented in this study come from computational simulations that can be reproduced by using the code available at https://github.com/MarioTovarCalonge/NC_Tovar_Arregui_codes

## Code availability

Code with the implementation of the novel methods introduced in this study is available at https://github.com/MarioTovarCalonge/NC_Tovar_Arregui_codesThose include algorithms written in Python (tested in version 3.6.7 2018-10-20), and R (tested in version 3.4.4 2018-03-15), with dependences to the following specific libraries: scipy, from Python, and bbmle^30^ and OIsurv^27^, from R. Scipy was used to implement Brent’s numerical method used to solve numerically the mathematical constraints between vaccine mechanisms and efficacy readouts. bbmle to run the maximum-likelihood models was used to estimate within-cohort rates of transition to disease. OIsurv was used to run Cox regression methods.
