# Supplementary material for: Bridging the gap between efficacy trials and model-based impact evaluation for new tuberculosis vaccines
Source: Nat Commun. 2019 Nov 29;10:5457. doi: 10.1038/s41467-019-13387-9 (PMC6884451; doi:10.1038/s41467-019-13387-9)
Supplement: Supplementary file 1 — Supplementary Information [file 41467_2019_13387_MOESM1_ESM.pdf]

Bridging the gap between efficacy trials and model-based impact evaluation for new tuberculosis vaccines. Supplementary Text S1.

Mario Tovar[a,b], Sergio Arregui[a,b], Dessisslava Marinova[c,d], Carlos Martín[c,d,e], Joaquín Sanz[a,b,f,†], and Yamir Moreno[a,b,g,†]

[a]Institute for Biocomputation and Physics of Complex Systems (BIFI), University of Zaragoza, Spain
[b]Department of Theoretical Physics, University of Zaragoza, Spain
[c]Department of Microbiology, Faculty of Medicine, University of Zaragoza, Spain
[d]CIBER Enfermedades Respiratorias, Instituto de Salud Carlos III, Madrid, Spain
[e]Service of Microbiology, Miguel Servet Hospital, Aragón, Spain
[f]Department of Medicine, Genetics Section, University of Chicago, IL, USA.
[g]ISI Foundation, Turin, Italy
[†]These authors contributed equally to this work

Contents

Supplementary Methods **2**
Module I: *In-silico* simulations of clinical trials. . . . 2
Agent-based stochastic model to simulate efficacy trials conducted in IGRA-negative cohorts for testing TB vaccines . . . 2
Relative contribution of different paths to disease in total TB incidence for trials conducted in IGRA-negative individuals. . . . 3
Module II: Data analysis of trials outcomes . . . 3
POI and POD efficacy readouts for trials conducted in IGRA-negative populations: $\mathrm{VE}_{\mathrm{inf}}$ and $\mathrm{VE}_{\mathrm{dis}}$ . . . 3
Mathematical constraints between efficacy readouts and vaccine mechanisms: estimation of ε_{p} . . 4
Module III. Impact evaluations of TB vaccines . . . 7
M.tb. transmission dynamics within age strata. . . . 7
Population dynamics across age strata: individuals' aging and demographic evolution. . . . 8
Vaccine descriptions . . . 8
Impact evaluation and uncertainty estimates . . . 9

Supplementary Figures **9**

Supplementary Methods

Mirroring the schematics of main text figure 2C, here we document supplementary details for the Methods within each of the three modules of our computational pipeline.

First, we discuss in detail the specifics of our agent-based model to simulate stochastically trial realizations (pipeline Module I: trials simulation), conducted on IGRA-negative cohorts. Here, we present simulations to corroborate the hypothesis that in this type of trials the vast majority of TB cases observed correspond to fast progression after a first infection event for follow up periods of up to 10 years.

Second, we describe in detail the different parts of our analytical approach to characterize vaccines from trials' data (pipeline Module II: vaccine characterization). This includes a discussion about the approach used to estimate $\mathrm{VE_{dis}}$ from the ratio of TB cases observed across cohorts at the end of the study, as well as the analytical resolution of the system of ordinary differential equations that lead to the analytical relationships $\mathrm{VE_{dis}} = f(\varepsilon_\beta, \varepsilon_\mathrm{r}, \varepsilon_\mathrm{p})$, for the case of IGRA-negative trials, and its equivalent $\mathrm{VE_{dis}} = f(F_o, \varepsilon_\mathrm{r}, \hat{\varepsilon}_\mathrm{p})$, for studies conducted on IGRA-positive individuals.

Third, we describe in further detail the *M.tb.*transmission model used to estimate vaccines' impact at larger scales, (pipeline module III: impact evaluation) which was calibrated and ran in Ethiopia, where the impact of the different vaccines studied was estimated as the number of TB cases prevented upon the introduction of the vaccine at the end of 2025, between that moment and the end of 2050.

Module I: *In-silico* simulations of clinical trials.

Agent-based stochastic model to simulate efficacy trials conducted in IGRA-negative cohorts for testing TB vaccines

In this work, we present a modeling framework to conduct *in silico* simulations of randomized clinical trials of vaccine efficacy. Our approach is based on an agent-based, stochastic model that simulates individually the evolution of each enrolled individual across disease states during the trial. The model follows a probabilistic scheme, whereby the individuals' fates during the trial are decided according to the epidemiological parameters, through a series of multinomial trials that are conducted at every time point (once a day, in this work), until the follow-up period of the trial is concluded.

For the case of TB, as represented in figure 1A in the main text, we have four states (susceptible S, fast latency F, slow latency L and active disease D), and five different types of transitions between them ($S->L$, $S->F$, $L->F$, $L->D$ and $F-D$). The model defines the following set of transition rules for individuals in each state, where all the random numbers ϕ drawn in the context of the multinomial tests proceed from uniform distributions in the interval $[0,1]$:

Susceptible individuals. On a daily basis, individuals may get infected, with a daily probability equal to β. If they get infected, they can either enter into fast latency with probability p, or slow latency, with probability $(1-p)$.

- 1. A first random number is drawn to decide if infection occurs: ϕ_1:
  - If $\phi_1 \leq \beta$, the individual gets infected, then:
    - A second random number is drawn to decide if fast progression occurs: ϕ_2:
      - If $\phi_2 \leq p$ individual enters into fast latency: **transition** $S->F$
      - If $\phi_2 > p$: individual develops LTBI: **transition** $S->L$
  - If $\phi > \beta$: individual does not get infected: **No transition**

LTBI individuals. Once an individual is in the LTBI reservoir (slow latency: L), he/she can either undergo endogenous reactivation (with daily probability r_L, or TB after a re-infection event (i.e. transition from L to F), with a daily probability equal to the product βpq (i.e. the probability of being infected times the probability of progressing to fast latency after that, modified by q, the protection factor that LTBI confers against progression to disease upon re-infection[1])

- 1. A random number is drawn ϕ_1:
  - If $\phi_1 \leq (\beta pq)$: individual will develop TB fast after exogenous re-infection: **transition** $L->F$
  - If $(\beta pq) < \phi_1 < (\beta pq + r_\mathrm{L})$: individual develops TB upon endogenous reactivation: **transition** $L->D$
  - If $(\beta pq + r_\mathrm{L}) < \phi_1$: individual remains LTBI: **No transition**

Individuals in F From the reservoir F, we only consider the transition to disease, with daily probability equal to r, as the only possible status change:

- 1. A random number is drawn to decide whether the transition to disease takes place ϕ_1:
  - If $\phi_1 \leq r$: individual develops TB: **transition** $F->D$
  - If $\phi_1 > r$: individual remains in F: **No transition**

The epidemiological parameters ruling the stochastic dynamics of the system ($\beta, p, q, r_{\rm L}$ and r) are re-scaled from their values reported in the main text to capture daily probabilities. Furthermore, each of these can in principle be modified by a vaccine, to simulate a vaccinated cohort, even if we only consider vaccines through a reduction of either β, p, or r in this study.

Relative contribution of different paths to disease in total TB incidence for trials conducted in IGRA-negative individuals.

One of the key assumptions of the methodology here proposed is that most TB cases observed within a trial conducted on IGRA-positive individuals are fast progressors after a first infection event. This assumption neglects the contribution to the overall pool of TB patients of endogenous reactivation from LTBI (since $r_{\rm L} << r$) and that of fast, progressive TB upon a re-infection event. Crucially, this assumption lies at the basis of our method to infer $\varepsilon_{\rm r}$ (see Materials and Methods section in the main text), by allowing us to consider that all the observations that we obtain in a trial for the transition times between infection and disease $t = t_{dis} - t_{inf}$ come from a unique distribution, (instead of an unknown mixture of several distributions), whose only parameter is the rate of fast progression to disease.

Considering that, we used our agent-based model to test the validity of this key assumption by tracking the particular paths to disease followed by each individual and see how often disease has been reached by fast progression, by slow-progression or after a reinfection event (Supplementary Figure 1A).

To do that, we perform 500 simulations of each cohort and we record the weight that every possible route has in each cohort, and average across realizations. We used the probabilistic parameters that are compatible with the reference case of the MVA85A study cohorts analyzed in the main text[2]:$\beta = 1.84 \cdot 10^{-4}$ d^{-1}, $r_{\rm L} = 2.05 \cdot 10^{-6}$ d^{-1} and $r = 2.66 \cdot 10^{-3}$ d^{-1}, which are rescaled to represent daily probabilities of infection, and fast/slow transition to disease, per day. Concerning $p = 0.375$, and $q = 0.21$, these share the same values, since they are probabilities by construction.

The results of this exercise are represented in Supplementary Figure 1B, in a control cohort (left), or in the intervention cohort of two vaccines associated with protection readouts of $\text{VE}_{\rm dis} = 50\%$ observed in a 4 years trial. The center panel corresponds to a vaccine conferring POD through a reduction in the probability of fast progression upon infection ($\varepsilon_{\rm p} = 0.5$), while the right panel corresponds to its counterpart based on reducing the fast progression rate to TB ($\varepsilon_{\rm r} = 0.74$). In all the cases explored in Supplementary Figure 1B, for different trial sizes and active mechanisms, a minimum of 93% of the individuals that develop active TB came from fast-progression, and less than 2% and 5% made its way through slow progression and reinfection, respectively.

As a consequence, our method works robustly in spite of vaccine mechanism or the duration of the study. As we see in Supplementary Figure 1C, this translates into estimations of the vaccine parameters ($\varepsilon_\beta, \varepsilon_{\rm r}, \varepsilon_{\rm p}$) that lie, in average, only 0.027 standard deviations away from the ground-truth values (always under 0.141 s.d.s).

Module II: Data analysis of trials outcomes

POI and POD efficacy readouts for trials conducted in IGRA-negative populations: $\text{VE}_{\rm inf}$ and $\text{VE}_{\rm dis}$

In order to estimate ε_β, we implement the Cox Regression Model[3], since the dynamics of vaccine-mediated prevention of infection is trivially compatible with the premises of Cox Regression Model (i.e. the infection Hazards in the control and vaccinated cohorts are proportional, regardless of time). However, that is not the case for efficacy against disease, where the applicability of Cox Regression Model is not guaranteed since the assumption of proportional hazards might not be correct depending on the features of the vaccine considered. To illustrate this situation, we have conducted Schoenfeld's Residual tests[4] to evaluate the accuracy of the proportional hazards hypothesis for different vaccines, characterized by different values of $\varepsilon_{\rm p}$ and $\varepsilon_{\rm r}$. The results of such test are represented in Supplementary Figure 2 panel A, where we show the proportion of tests with a p-value lower than 0.05 where the hypothesis of proportional hazards can be rejected (under the null hypothesis the proportion should be precisely 5%) . As a conclusion, we see that vaccines that delay the fast progression rates violate more often the hypothesis of Hazards Proportionality, well above 5%, which is coherent

with the observation shown in Supplementary Figure 2, panel B, where we see how the observed efficacies of an ε_r-based vaccine are strongly dependent on time.

Motivated by that limitation of classical survival analysis regarding $\mathrm{VE}_{\mathrm{dis}}$ we decided to make use of a more elementary estimate for measuring the efficacy of a vaccine against disease, namely $\mathrm{VE}_{\mathrm{dis}}(T) = 1 - \rho(T)$, where $\rho(T)$ is the disease ratio evaluated at the end of the trial, as defined in equation 36 of the main text.

This alternative way to estimate $\mathrm{VE}_{\mathrm{dis}}$ (or, equivalently, ρ) enables a tractable analytical relationship between ρ, and the triad ($\varepsilon_\beta \equiv \mathrm{VE}_{\mathrm{inf}}, \varepsilon_r, \varepsilon_p$), which allows us to estimate the last one from the measurements of the other three. Additionally, even though the proportional hazards hypothesis is not perfectly met for all vaccines, it is worth noticing that the differences observed between proportion-based and Cox-regression measures of $\mathrm{VE}_{\mathrm{dis}}(T)$ are limited.

In Supplementary Figure 2C we compared the results obtained with both measurements of efficacy at disease for different vaccines, observing that their relative differences remain below 8% of the mean of the efficacies estimated from both methods.

Mathematical constraints between efficacy readouts and vaccine mechanisms: estimation of ε_p

IGRA-negative cohorts: derivation of the relation $\mathbf{VE}_{\mathbf{dis}} = f(\varepsilon_\beta, \varepsilon_p, \varepsilon_r)$ According to the scheme of transitions that we use to model a clinical trial (main text figure 1A), the evolution of the four states (S: Susceptible, F: Fast-progressors, L: Slow-progressors, latent infection and D: Disease) is given, in the control cohort (subindex c), by the following differential equations:

$$\frac{dS_c(t)}{dt} = -\beta S_c(t) \tag{1}$$

$$\frac{dF_c(t)}{dt} = \beta p S_c(t) - rF_c(t) + \beta pq L_c(t) \tag{2}$$

$$\frac{dL_c(t)}{dt} = \beta(1-p)S_c(t) - r_L L_c(t) - \beta pq L_c(t) \tag{3}$$

$$\frac{dD_c(t)}{dt} = rF_c(t) + r_L L_c(t) \tag{4}$$

For the vaccinated cohort (subindex v), the parameters β, p and r are modified by the action of the vaccine:

$$\frac{dS_v(t)}{dt} = -(1-\varepsilon_\beta)\beta S_v(t) \tag{5}$$

$$\frac{dF_v(t)}{dt} = (1-\varepsilon_\beta)\beta(1-\varepsilon_p)pS_v(t) - (1-\varepsilon_r)rF_v(t) + (1-\varepsilon_\beta)\beta(1-\varepsilon_p)pqL_v(t) \tag{6}$$

$$\frac{dL_v(t)}{dt} = (1-\varepsilon_\beta)\beta(1-(1-\varepsilon_p)p)S_v(t) - r_L L_v(t) - (1-\varepsilon_\beta)\beta(1-\varepsilon_p)pqL_v(t) \tag{7}$$

$$\frac{dD_v(t)}{dt} = (1-\varepsilon_r)rF_v(t) + r_L L_v(t) \tag{8}$$

Solving the system of differential equations, we obtain the proportion for every state at each cohort. Initial conditions are $S_x = 1$ and $L_x = F_x = D_x = 0$ for $x = c, v$, i.e. all individuals are susceptible at the beginning of the trial.

$$S_c(t) = \exp(-\beta t) \tag{9}$$

$$S_v(t) = \exp(-(1-\varepsilon_\beta)\beta t) \tag{10}$$

$$F_c(t) = K\exp(-rt) + \frac{\beta p}{r-\beta}\exp(-\beta t) + \frac{\beta^2 pq(1-p)}{r_L + \beta pq - \beta}\left[\frac{\exp(-\beta t)}{r-\beta} - \frac{\exp(-(r_L+\beta pq)t)}{r - r_L - \beta pq}\right] \tag{11}$$

where:

$$K = -\beta p\left[\frac{r - r_L - q\beta}{(r-\beta)(r-r_L-\beta pq)}\right] \tag{12}$$

$$F_{\rm v}(t) = K' \exp(-(1-\varepsilon_{\rm r})rt) + \frac{(1-\varepsilon_\beta)\beta(1-\varepsilon_{\rm p})p}{(1-\varepsilon_{\rm r})r-(1-\varepsilon_\beta)\beta}\exp(-(1-\varepsilon_\beta)\beta t)$$
$$+\frac{((1-\varepsilon_\beta)\beta)^2(1-\varepsilon_{\rm p})pq(1-(1-\varepsilon_{\rm p})p)}{r_{\rm L}+(1-\varepsilon_\beta)\beta(1-\varepsilon_{\rm p})pq-(1-\varepsilon_\beta)\beta}\left[\frac{\exp(-(1-\varepsilon_\beta)\beta t)}{(1-\varepsilon_{\rm r})r-(1-\varepsilon_\beta)\beta}\right.$$
$$\left.-\frac{\exp(-(r_{\rm L}+(1-\varepsilon_\beta)\beta(1-\varepsilon_{\rm p})pq)t)}{(1-\varepsilon_{\rm r})r-r_{\rm L}-(1-\varepsilon_\beta)\beta(1-\varepsilon_{\rm p})pq}\right] \tag{13}$$

where:

$$K' = -(1-\varepsilon_\beta)\beta(1-\varepsilon_{\rm p})p\left[\frac{(1-\varepsilon_{\rm r})r-r_{\rm L}-(1-\varepsilon_\beta)\beta(1-\varepsilon_{\rm p})pq-q(1-\varepsilon_\beta)\beta(1-(1-\varepsilon_{\rm p})p)}{((1-\varepsilon_{\rm r})r-(1-\varepsilon_\beta)\beta)((1-\varepsilon_{\rm r})r-r_{\rm L}-(1-\varepsilon_\beta)\beta(1-\varepsilon_{\rm p})pq)}\right] \tag{14}$$

$$L_{\rm c}(t) = \frac{\beta(1-p)}{r_{\rm L}+\beta pq-\beta}\left[\exp(-\beta t)-\exp(-(r_{\rm L}+\beta pq)t)\right] \tag{15}$$

$$L_{\rm v}(t) = \frac{(1-\varepsilon_\beta)\beta(1-(1-\varepsilon_{\rm p})p)}{r_{\rm L}+(1-\varepsilon_\beta)\beta(1-\varepsilon_{\rm p})pq-(1-\varepsilon_\beta)\beta}[\exp(-(1-\varepsilon_\beta)\beta t)-\exp(-(r_{\rm L}+(1-\varepsilon_\beta)\beta(1-\varepsilon_{\rm p})pq)t)] \tag{16}$$

$$D_{\rm c}(t) = 1-S_{\rm c}(t)-L_{\rm c}(t)-F_{\rm c}(t) = 1-\alpha\exp(-\beta t)+\Omega\exp(-rt)+\gamma\exp(-(r_{\rm L}+\beta pq)t) \tag{17}$$

where:

$$\begin{aligned}\alpha &= rr_{\rm L}+\beta pqr-\beta r_{\rm L}-\beta pr+\beta pr_{\rm L}\\ \Omega &= \beta p\frac{r-r_{\rm L}-q\beta}{(r-\beta)(r-r_{\rm L}-\beta pq)}\\ \gamma &= \beta r-\beta r_{\rm L}-\beta pr+\beta pr_{\rm L}\end{aligned} \tag{18}$$

$$D_{\rm v}(t) = 1-\alpha'\exp(-(1-\varepsilon_\beta)\beta t)+\Omega'\exp(-(1-\varepsilon_{\rm r})rt)+\gamma'\exp(-(r_{\rm L}+(1-\varepsilon_\beta)\beta(1-\varepsilon_{\rm p})pq)t) \tag{19}$$

where:

$$\begin{aligned}\alpha' &= (1-\varepsilon_{\rm r})rr_{\rm L}+(1-\varepsilon_\beta)\beta[(1-\varepsilon_{\rm p})pq(1-\varepsilon_{\rm r})r-r_{\rm L}-(1-\varepsilon_{\rm p})p(1-\varepsilon_{\rm r})r+(1-\varepsilon_{\rm p})pr_{\rm L}]\\ \Omega' &= (1-\varepsilon_\beta)\beta p\frac{(1-\varepsilon_{\rm r})r-r_{\rm L}-q(1-\varepsilon_\beta)\beta}{((1-\varepsilon_{\rm r})r-(1-\varepsilon_\beta)\beta)((1-\varepsilon_{\rm r})r-r_{\rm L}-(1-\varepsilon_\beta)\beta(1-\varepsilon_{\rm p})pq)}\\ \gamma' &= \beta[(1-\varepsilon_{\rm r})r-r_{\rm L}-(1-\varepsilon_{\rm p})p(1-\varepsilon_{\rm r})r+(1-\varepsilon_{\rm p})pr_{\rm L}]\end{aligned} \tag{20}$$

Then, we obtain the disease-ratio at the end of the trial as follows:

$$\rho(T) = \frac{D_{\rm v}(T)}{D_{\rm c}(T)} = \frac{[1-\alpha' e^{-(1-\varepsilon_\beta)\beta T}+\Omega' e^{-(1-\varepsilon_{\rm r})rT}+\gamma' e^{-(r_{\rm L}+(1-\varepsilon_\beta)\beta(1-\varepsilon_{\rm p})pq)T}]}{[1-\alpha e^{-\beta T}+\Omega e^{-rT}+\gamma e^{-(r_{\rm L}+\beta pq)T}]} \tag{21}$$

The ratio will depend not only on the parameters of the vaccine (ε_β, $\varepsilon_{\rm p}$ and $\varepsilon_{\rm r}$) but also on the natural parameters of the disease (β, p, q, r and $r_{\rm L}$) and on the trial follow-up period T. This expression for the disease ratio ρ as a function of ε_β, $\varepsilon_{\rm p}$ and $\varepsilon_{\rm r}$ captures in itself the relation that we looked for, namely, $\mathrm{VE_{dis}} = f(\varepsilon_\beta, \varepsilon_{\rm p}, \varepsilon_{\rm r})$, since $\mathrm{VE_{dis}}$ is estimated as $1-\rho$.

IGRA-negative trials: estimation of $\varepsilon_{\rm p}$ Equation 21 defines the functional relationship $\rho = f(\varepsilon_\beta, \varepsilon_{\rm p}, \varepsilon_{\rm r})$ that binds together the two vaccine efficacy readouts ($\mathrm{VE_{dis}} = 1-\rho$ and $\mathrm{VE_{inf}} \equiv \varepsilon_\beta$) to $\varepsilon_{\rm r}$ and the last unknown parameter, namely, $\varepsilon_{\rm p}$. Therefore, from eq. 21, we can solve for this last parameter, if not explicitly, by numerical means, once we have estimated the other three. Specifically, we use the classic Brent method included in python's scipy package (scipy.optimize.brentq from Scipy v1.3.0).

In order to estimate $\varepsilon_{\rm p}$ uncertainty, we propagate standard error from ε_β (Cox regression), $\varepsilon_{\rm r}$ (from Maximum likelihood-based inference of fast-progression rates at control cohort and at vaccine cohort, as explained in the main text) and ρ. Regarding the last source of uncertainty, we obtain the variance associated with the disease-fraction as follows:

$$s^2 = \frac{1-D_{\rm c}(T)}{D_{\rm c}(T)N}+\frac{1-D_{\rm v}(T)}{D_{\rm v}(T)N} \tag{22}$$

which yields the following confidence interval:

$$\mathrm{CI} = 1 - \exp\left(\ln\left(\frac{D_\mathrm{v}(T)}{D_\mathrm{c}(T)}\right) \mp z_{1-\frac{\alpha}{2}} s\right) \tag{23}$$

where $D_\mathrm{c}(T)$ and $D_\mathrm{v}(T)$ are the fraction of cases observed at both cohorts during the follow-up period, and $z_{1-\frac{\alpha}{2}}$ is the standard score for the chosen level of significance α (95%).

Once the estimates of the three parameters ρ, $\mathrm{VE_{inf}} \equiv \varepsilon_\beta$ and ε_r and their uncertainties are available, we use equation 21 to generate the estimate of ε_p and its uncertainty as follows. In a series of N=1000 realizations, we obtain estimates for each of the three known parameters that are drawn for a normal distribution with parameters coherent with mean and CI estimates for that parameter. Doing this independently for the three parameters, we obtain a distribution of corresponding values for ε_p, from which to derive a confidence interval.

Trials conducted on IGRA-positive cohorts In the case of trials conducted on IGRA-positive subjects, (figure 4A in the main text), the evolution of the three states in the control cohort (subindex c) is given by:

$$\frac{dF_\mathrm{c}(t)}{dt} = -rF_\mathrm{c}(t) + \beta pq L_\mathrm{c}(t) \tag{24}$$

$$\frac{dL_\mathrm{c}(t)}{dt} = -r_\mathrm{L} L_\mathrm{c}(t) - \beta pq L_\mathrm{c}(t) \tag{25}$$

$$\frac{dD_\mathrm{c}(t)}{dt} = rF_\mathrm{c}(t) + r_\mathrm{L} L_\mathrm{c}(t) \tag{26}$$

For the vaccinated cohort (sub-index v), reinfections rate (βpq) and parameter r are modified by the action of the vaccine:

$$\frac{dF_\mathrm{v}(t)}{dt} = -(1-\varepsilon_\mathrm{r})rF_\mathrm{v}(t) + (1-\hat{\varepsilon}_\mathrm{p})\beta pq L_\mathrm{v}(t) \tag{27}$$

$$\frac{dL_\mathrm{v}(t)}{dt} = -r_\mathrm{L} L_\mathrm{v}(t) - (1-\hat{\varepsilon}_\mathrm{p})\beta pq L_\mathrm{v}(t) \tag{28}$$

$$\frac{dD_\mathrm{v}(t)}{dt} = (1-\varepsilon_\mathrm{r})rF_\mathrm{v}(t) + r_\mathrm{L} L_\mathrm{v}(t) \tag{29}$$

Solving the system of differential equations, we obtain the proportion for every state at each cohort. One crucial difference between this case and the previous one is that, in the former case, all N individuals recruited begin the trial within the state S, but now, they are distributed between states L and F as follows: $L_x(t=0) = L_0$, and $F_x(t=0) = N - F_0$ for $x = c, v$. This introduces one additional unknown parameter, namely, the number of individuals F_0 that begin the trial within the state F in each cohort, even assuming that F_0 has the same value in both of them. The results are as follow:

$$F_\mathrm{c}(t) = F_0 \exp(-rt) + \frac{\beta pq L_0}{r - r_\mathrm{L} - \beta pq}\left[\exp(-(r_\mathrm{L} + \beta pq)t) - \exp(-rt)\right] \tag{30}$$

$$F_\mathrm{v}(t) = F_0 \exp(-(1-\varepsilon_\mathrm{r})rt) + \frac{(1-\varepsilon)\beta pq L_0}{(1-\varepsilon_\mathrm{r})r - r_\mathrm{L} - (1-\varepsilon)\beta pq}\left[\exp(-(r_\mathrm{L} + (1-\varepsilon)\beta pq)t) - \exp(-(1-\varepsilon_\mathrm{r})rt)\right] \tag{31}$$

$$L_\mathrm{c}(t) = L_0 \exp(-(r_\mathrm{L} + \beta pq)t) \tag{32}$$

$$L_\mathrm{v}(t) = L_0 \exp(-(r_\mathrm{L} + (1-\varepsilon)\beta pq)t) \tag{33}$$

$$\begin{aligned} D_\mathrm{c}(t) = \quad & N_0 - L_\mathrm{c}(t) - F_\mathrm{c}(t) = N_0 - L_0 \exp(-(r_\mathrm{L} + \beta pq)t) - F_0 \exp(-rt) \\ & + \frac{\beta pq L_0}{r - r_\mathrm{L} - \beta pq}\left[\exp(-(r_\mathrm{L} + \beta pq)t) - \exp(-rt)\right] \end{aligned} \tag{34}$$

$$\begin{aligned} D_\mathrm{v}(t) = & N_0 - L_\mathrm{v}(t) - F_\mathrm{v}(t) = N_0 - L_0 \exp(-(r_\mathrm{L} + (1-\varepsilon)\beta pq)t) \\ & - F_0 \exp(-(1-\varepsilon_\mathrm{r})rt) + \\ & \frac{(1-\varepsilon)\beta pq L_0}{(1-\varepsilon_\mathrm{r})r - r_\mathrm{L} - (1-\varepsilon)\beta pq}\left[\exp(-(r_\mathrm{L} + (1-\varepsilon)\beta pq)t) - \exp(-(1-\varepsilon_\mathrm{r})rt)\right] \end{aligned} \tag{35}$$

From these expressions, we obtain the disease-ratio at the end of the trial as follows:

$$\rho(T) = \frac{D_{\mathrm{v}}(T)}{D_{\mathrm{c}}(T)} \tag{36}$$

Again, this ratio depends not only on the parameters of the vaccine ($\hat{\varepsilon}_{\mathrm{p}}$ and ε_{r}) but also on the natural parameters of the disease (β, p, q, r, and r_{L}) and on the trial follow-up period T.

But unlike the previous case, equation 36 defines a functional relation $\rho = f(F_0, \varepsilon_{\mathrm{r}}, \hat{\varepsilon}_{\mathrm{p}})$ that does not allow to solve for $\hat{\varepsilon}_{\mathrm{p}}$. This is because of two reasons. On the one hand, we do not know or have any means to estimate the initial fraction of fast progressors F_0. On the other hand, now, we cannot estimate independently ε_{r}, since we do not know the IGRA-conversion times, and therefore we cannot observe the times between infection and disease. As a result, we can only derive, using numerical solvers (Brent method included in the scipy package (scipy.optimize.brentq from Scipy v1.3.0), in Python), the relation that is established between ε_{r} and $\hat{\varepsilon}_p$, for different levels of F_0, and different observations of $\mathrm{VE}_{\mathrm{dis}} = 1 - \rho$ (figure 4B in the main text).

Module III. Impact evaluations of TB vaccines

In this work, we applied the model described in[5] to estimate the long-term impacts of different vaccines, in terms of their ability to reduce the total number of incident cases upon their introduction in a large scale setting.

This tool is a deterministic, age-structured model based on ordinary differential equations, where individuals belonging to different age-strata are considered to experiment different levels of epidemiological risk that translate into parameter values that, in general, depend on age. According to our model, the dynamical architecture that defines the disease dynamics within each age-group is similar to previous approaches. Other aspects, such as the description of the contact patterns among age-groups that lead to pathogen's spreading, or the coupling between populations' aging and transmission dynamics introduce important novelties with respect to previous literature, as profusely described in[5], where the reader is referred to for further details.

Using this model, we simulated the introduction of different vaccines in a country such as Ethiopia, at the end of 2025, and evaluated their impacts as the number of TB cases prevented by the vaccine until the end of 2050.

In the following lines we summarize the main aspects of the model used to evaluate vaccines' impact, including its Natural History description of the disease, the description of the coupling between populations' aging and disease dynamics, the generalization undertaken in this study to describe the introduction of vaccines, and the propagation of the uncertainty incurred in vaccine characterization to the impact estimates. The entire model is described at a much greater level of detail in the original publication[5].

M.tb. transmission dynamics within age strata.

The model we use to estimate vaccines impact is age-structured, including 15 different age groups, 14 of them covering 5 years of age up to 70 years old, and the last one containing all individuals older than 70 years old. Within each age group, we distinguish two branches of individuals: vaccinated and non-vaccinated (see Supplementary Figure 3A). Within each of these branches, we have a class of unexposed individuals –susceptible–, two different infection paths to disease –fast and slow – and six different kinds of disease, depending on its aetiology: -non pulmonary, pulmonary (smear positive) and pulmonary (smear negative)–, and depending on if it is untreated or treated. Right after the disease phase, we explicitly consider the treatment outcomes contemplated by the WHO data schemes: treatment completion (or success), default, failure, and death[6].

As a summary, there are several types of transitions possible between the states represented in Supplementary Figure 3A:

- Infection processes: after contact with an infectious individual, susceptible individuals (S) get infected, entering either the fast (F), or slow latency states (L).
- Re-infection processes: individuals in the slow latency reservoir can get re-infected, a fraction of which will develop TB fast after re-infection. This is modelled as a transition from L to F.
- Development of active TB: infected individuals (either F or L) may develop initially undiagnosed -and thus untreated- TB (D).
- TB diagnosis: with some delay after the disease onset, TB gets diagnosed and treatment starts (transition D to T)
- Treatment outcomes: (transitions from T to R) different possible outcomes are possible: –either success or failure/default-

- Disease relapse: (transitions back from T to R)
- Death: active TB patients, either diagnosed or not, are assigned with a TB specific mortality rate.

The specific details about the mathematical parameterization of these and other dynamical transitions are explicitly enumerated in the supplementary appendix of[5]. These rules define how the fraction of individuals in each of the different disease states evolve with time in each age group. From this information, TB incidence, prevalence of infection and disease, and mortality levels can be estimated within each age group, as well as in the entire population.

According to the model, infections occur after contact between susceptible individuals and infectious ones. If $S(a,t)$ represents the number of susceptible subjects in the age group a, at a given moment t, the number of new infections that will be observed will be equal to the product of $S(a,t)$ and the force of infection perceived by that sub-population, $\lambda(a,t)$, which represents the fraction of susceptible individuals who get infected per year. In turn, the force of infection is proportional to the following sum:

$$\sum_{a'} \xi_c(a,a',t)\Upsilon(a',t) \tag{37}$$

where $\Upsilon(a',t)$ is the density of all the infectious individuals within age-group a' at time step t, weighted by their relative infectiousness; and $\xi_c(a,a',t)$ represents the relative contact frequency that an individual of age a has with individuals of age a' at time t, with respect to the overall average of contacts that an individual has per unit time with anyone else.

For the computation of the contact matrices used in our model, we have integrated data from different survey studies conducted in African countries (Kenya[7], Zimbabwe[8] and Uganda[9]), to obtain a unique matrix broadly representative of contact structures in Africa, as described in[5,10]. Importantly, we also take into account that, as the demographic structure of the population changes, the contact patterns change too[10]. Even if data availability is limited, and thus the contact matrix used is an approximation of the situation in Ethiopia, our approach constitutes an improvement with respect to classical models[11] that assume that contact patterns are homogeneously distributed across age-groups. That simplification, which we avoid here, is incompatible with surveys data[12], and has a direct impact in model forecasts[5].

Population dynamics across age strata: individuals' aging and demographic evolution.

This model, unlike the previous tools described in sections and , includes the simultaneous description of the disease dynamics across all age-groups in an entire population, using parameters that are, in general, dependent on individuals' age. Therefore, it is not enough to describe how the sub-populations associated with the disease states evolve in time, but the model also includes an aging dynamics whereby individuals transit across the different age-strata as they get older (Supplementary Figure 3B). Finally, two additional ingredients that are key to describe populations' aging are also included in our model. First, we consider empiric data and forecasts to model past and future fertility levels, respectively[13] . Second, we introduce continuous correction terms $\Delta_N(a,t)$ that are added or subtracted from the population within the age stratum a at time t while the simulation unfolds. These terms are calculated dynamically to make the time evolution of the demographic pyramid match the demographic forecasts reported in the United Nations population division database until 2050[13]. This way, the correction terms $\Delta_N(a,t)$ are distributed among all disease states proportionally to their relative size. These terms represent changes in the population of each stratum that are unrelated to the dynamics of the disease (TB unrelated mortality and migratory fluxes).

Vaccine descriptions

In the work presented in[5] the model is not used to evaluate the impact of vaccines. Thus we need to extend the model by adding a second branch for vaccinated individuals. This second branch follows qualitatively the same Natural History, already explained in[5], but quantitatively the disease parameters that regulate all the transitions between states could be modified by the epidemiological intervention considered (in this case vaccines). Thus, for every parameter x in the non-vaccinated branch, we could have $x' = (1-\varepsilon_x)x$ in the vaccinated branch, where ε_x represents the effect of the vaccine over the parameter x.

Here we study vaccines that act over three different parameters: the infection rate, (ε_β), (which is a coefficient that modulates the force of infection of all age groups $\lambda(a,t)$ alike), the probability of fast progression to TB (ε_p) and the rate of fast progression itself (ε_r). We assume $\varepsilon_x = 0$ for every other parameter (i.e., the vaccine has no other effect). Individuals in the vaccinated branch contribute to the force of infection with the same weight of non-vaccinated individuals (i.e., a reduction in infectiousness or contact rate as a result of vaccination

is not considered).

Furthermore, two types of vaccination campaigns are considered, either focused on new-borns or adolescents. Newborn vaccination acts on the flux of new births, which, from the moment the vaccination campaign starts (end of 2025), is directly introduced within the vaccinated branch instead of the non-vaccinated. Therefore, we are not describing possible delays between birth and vaccination, no matter whether the new vaccine is applied instead of BCG, or in addition to it, where these delays might thus be larger. In what regards vaccines introduced to adolescents, we progressively vaccinate individuals without a history of past TB as they turn fifteen years old from the beginning of the campaign. This is modelled by the introduction of the red fluxes in Supplementary Figure 3A, that represent vaccination of susceptible and latently infected individuals. In both types of vaccination campaigns, the vaccine is introduced at the end of 2025 and remains active until the end of 2050, always acting on the same population targets (new-borns or fifteen-years-old individuals, who are vaccinated as they turn that age).

For simplicity, we have assumed 100% vaccine coverage, which is however not far away from the 92% level reached in Ethiopia in 2016[14]. Regarding vaccine protection, in the general case, we have assumed a stable, non-decaying profile. We have also conducted simulations where the diverse vaccine parameters ε decay at rates of 1% and 5% per year, modelled through the introduction of decaying protection levels across age grouops within the vaccinated cohort (i.e. no flux back to the non-vaccinated branch is included). As shown in Supplementary Figure 4, these patterns of immunity waning do not interfere with the main observation of this study, namely, the fact that impacts associated with vaccines that lean on different dynamical mechanisms to provide analogous readouts of $\mathrm{VE_{dis}}$ are significantly different when evaluated at larger demographic and temporal scales.

Impact evaluation and uncertainty estimates

To obtain vaccine impacts, we first calibrate the model, estimating the infectiousness β and the diagnosis rate d so as to reproduce overall incidence and mortality rates reported in the WHO TB database in Ethiopia in between 2000 and 2015. Once the model is calibrated within that period, we use it to produce forecasts until 2050 under two different scenarios: one scenario of no-intervention, and another one where a vaccine is introduced by the end of 2025. Then, we obtain impact estimates of the different vaccines analysed in this study as the difference in total TB cases between those two scenarios, for different vaccines tested.

All the input data sources that the model integrates to produce these impacts carry intrinsic uncertainties whose influence on model-based forecasts of vaccine impact are evaluated. To do that, we followed the same procedure described in[5], according to which the outcome of the model (i.e. the impact estimate) is evaluated under a set of alternative scenarios where all sources of uncertainty are evaluated, one by one, at the limits of their respective confidence intervals. This yields the estimation of deviation terms in model outcomes δ_i, each of which is associated with the change in the impact estimate that occurs in response to the uncertainty in the i-th model input. Importantly, some of the sources of uncertainty are multi-dimensional, meaning that affect several parameters whose uncertainty estimates are mutually correlated, and therefore need to be evaluated at their limits of their CIs at the same time.

The original model, as presented originally in[5] contemplates as many as 22 independent uncertainty sources whose effects on model's outcomes are propagated, including epidemiological parameters, demographic data, contact matrices, and initial TB incidence and mortality estimates (that condition model calibration, and therefore, model outcomes). To all these sources of uncertainty, that affect both the incidence levels to be modeled in the control and vaccine runs, we add now the uncertainties of the inferred vaccine parameters, which we parametrize introducing two additional sources of uncertainty. First, we add the uncertainty associated with the protection of infection provided by the vaccine through ε_{r} (POI), by evaluating this parameter at the limits of its C.I. Then, we evaluate the impact uncertainty coming from the estimation of the parameters that contribute to vaccine-mediated prevention of disease (POD): ε_{r} and ε_{p}. To do so in a simple way that takes into account the co-dependence between these parameters, we evaluate the model under the two extreme scenarios where one of the parameters takes the value corresponding to the upper limit of its C.I. while the other one moves to its lower limit, and vice-versa. These additional sources of model uncertainty are thus added to all others in order to estimate global confidence intervals obtained as the square root of the sum o their squares.

Supplementary Figures

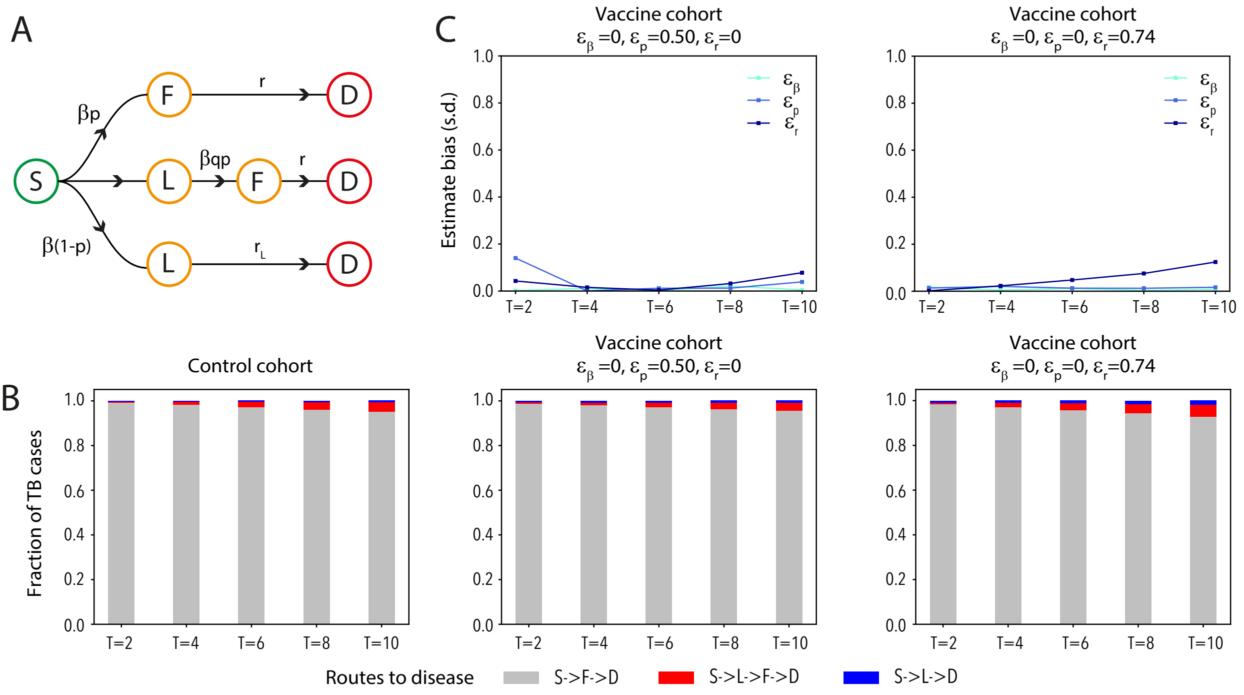

Supplementary figure 1. A. Schematics of the different routes to disease present in our model, which can be monitored for every single individual simulated within our agent-based framework. B. Fraction of TB cases that correspond to the three different paths to disease, for different trial durations, in a control cohort (left), a vaccine cohort based on the reduction of the probability of fast progression upon infection (center, $\varepsilon_\beta = \varepsilon_r = 0, \varepsilon_p = 0.5$) and a vaccine cohort based on the reduction of fast progression rates (right, $\varepsilon_\beta = \varepsilon_p = 0, \varepsilon_r = 0.74$). C. Bias incurred for different vaccine mechanisms and trial sizes in the estimates of the epsilon parameters (deviation between the median estimate of trials simulations and ground truth, scaled by the standard deviation associated with the estimated confidence interval).

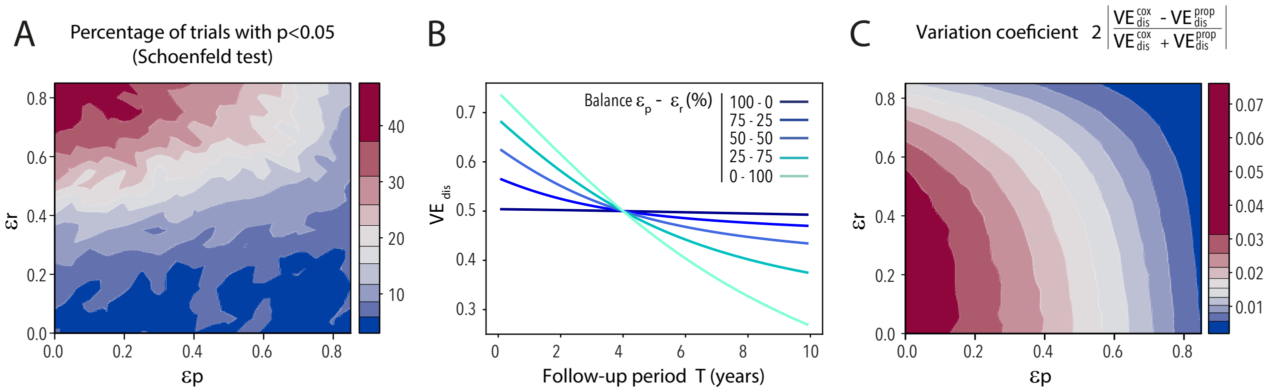

Supplementary figure 2. A. Proportion of Schoenfeld's Residual tests with a p-value below 5% as a function of ε_p and ε_r. Simulations have been performed with a Cohort Size of 3000 individuals, a follow-up period of 4 years, and 500 iterations. B. Evolution of measured VE_{dis} as a function of the follow-up period for five different vaccines that are compatible with a common readout from a $T = 4$ years trial but lean on different ratios $(\varepsilon_p, \varepsilon_r)$ in their mechanisms of action. Unlike vaccines that are based on reducing the probability of fast TB (whose VE_{dis} readouts are essentially independent of follow-up periods), the readout of VE_{dis} for vaccines that delay incubation periods is strongly dependent on trial duration. C. Coefficients of variation. Simulations have been performed with a Cohort Size of 3000 individuals and a follow-up period of 4 years.

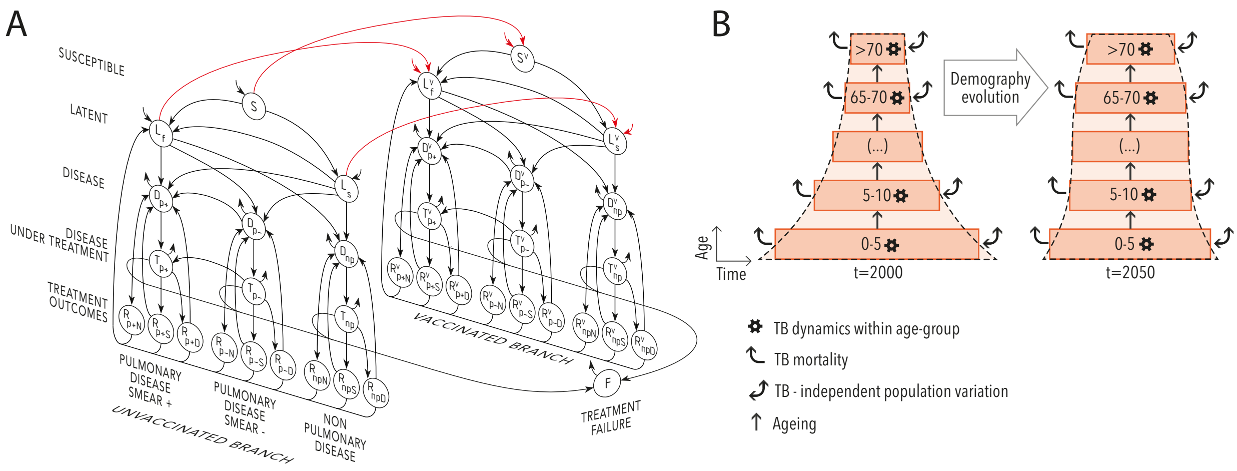

Supplementary figure 3. A. Natural History scheme of the TB spreading model. S: susceptible. L: latent. D: (untreated) disease, T (treated) disease, R recovered, F: failed recovery. Types of TB considered: p+: Pulmonary Smear-Positive, p-: Pulmonary Smear-Negative, np: Non-pulmonary. Treatment outcomes: RN: Natural recovery, RS: Successful treatment, RD: Default (abandon of treatment), F: treatment failure. To the control branch (i.e. non-vaccinated), a parallel branch where individuals have received a vaccine (i.e. red arrow transitions) is added. In the vaccinated branch, the dynamics is the same with the exception of that, depending on the vaccine type, the infectiousness, probabilities and/or rates of fast-progression to disease are reduced, as dictated by the vaccine descriptors ε_β, ε_p and ε_r, respectively. B. Schematic representation of population aging under our model. Besides the dynamics among the different disease states that occur within each age group, individuals get older, which is captured introducing upwards flows of individuals transiting the different strata of the population pyramid (vertical arrows). To control that the overall demographic pyramid evolves according to the UN population division's forecasts[13], additional correction terms are introduced, capturing population dynamics due to causes foreign to TB. Figure adapted from[5], where the reader is referred to for further technical specifications.

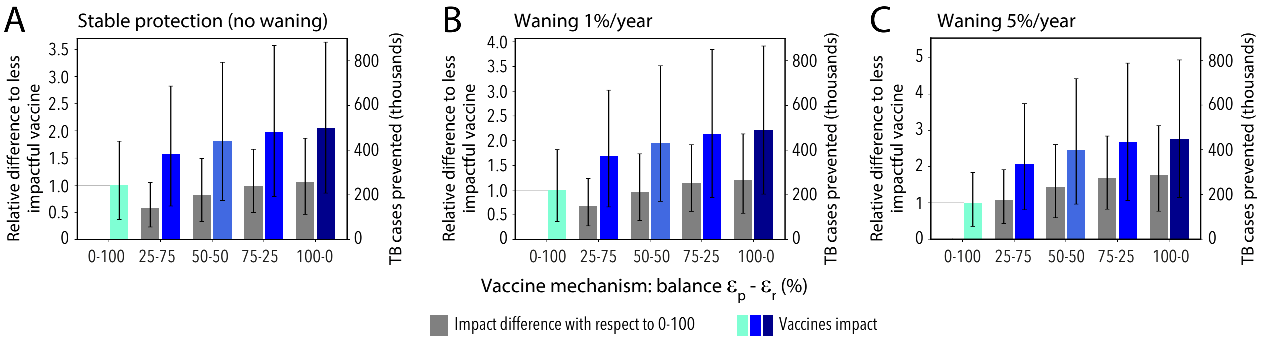

Supplementary figure 4. Impact foreseen for several POD vaccines characterized by different combinations of the initial values of their parameters ε_p-ε_r, under different scenarios regarding protection waning after vaccination. A: reference scenario: persistent protection. B: Moderate waning: ε_p and ε_r decay at a rate of 1% per year. C: Strong waning: they decay a 5% every year. Error bars (black bars) represent the 95% confidence interval.

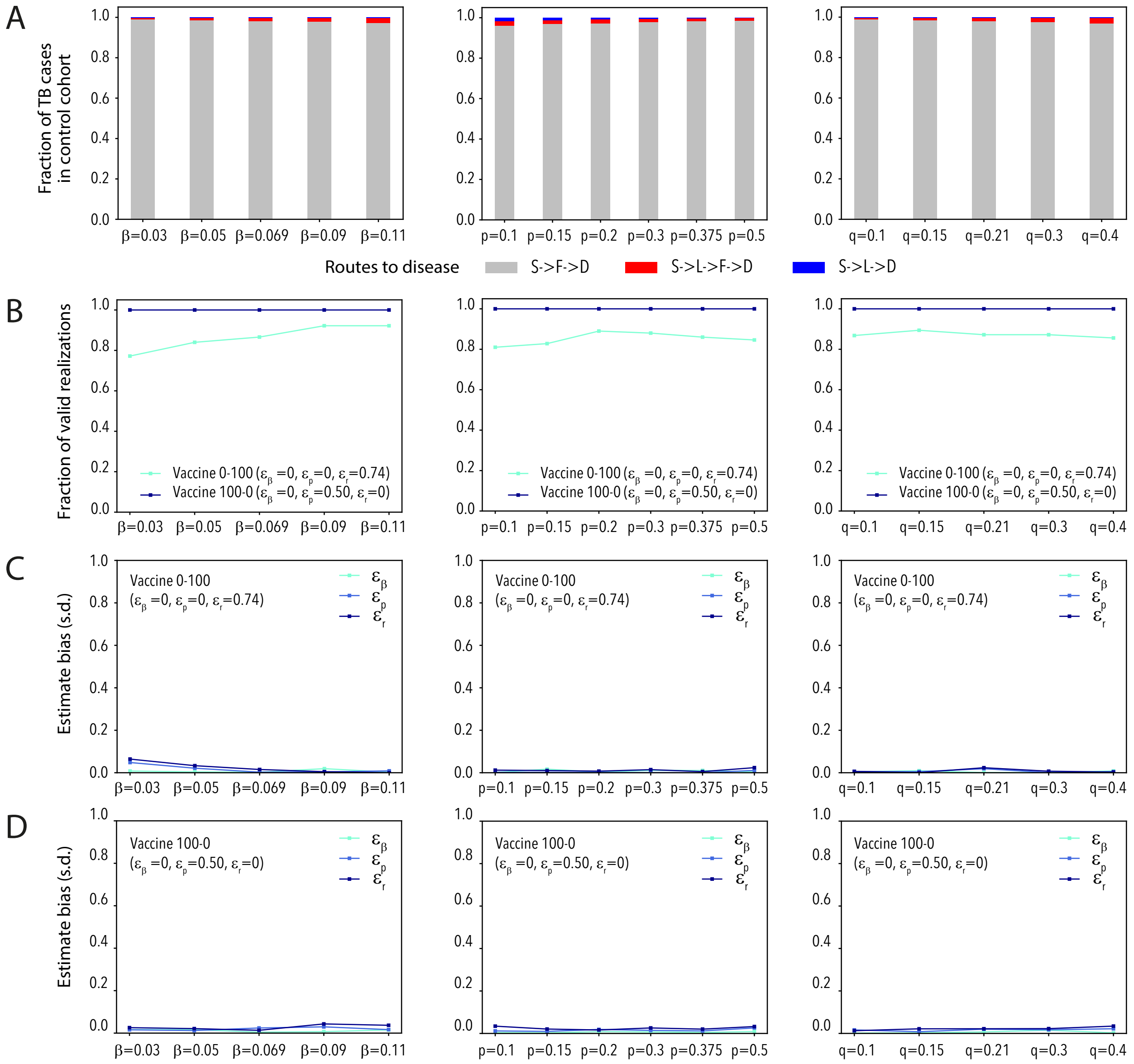

Supplementary figure 5. Method performance metrics evaluated under alternative scenarios, covering assorted levels of the infection rate ($\beta = (0.03, 0.05, 0.069, 0.09, 0.11)$, left column), different values for the probability of fast progression upon infection ($p = (0.1, 0.15, 0.2, 0.3, 0.375, 0.5)$, center column) and different levels of LTBI-associated protection against TB progression after secondary infection ($q = (0.1, 0.15, 0.21, 0.3, 0.4)$, right column). Trial duration and cohort sizes are left as in the main text ($T = 4$ years) ($N = 3000$ individuals). In each case we register the fraction of TB cases associated with the different routes to disease present in our model (A, top row), the fraction of simulated trials leading to valid vaccine characterizations (B, second row), the bias incurred in the estimation of vaccine parameters for a reference vaccine delaying fast progression ($\varepsilon_{\mathrm{r}} = 0.74$, and $\varepsilon_{\beta} = \varepsilon_{\mathrm{p}} = 0$, third row, C), and for a conjugate vaccine acting through a reduction of the probability of fast progression ($\varepsilon_{\mathrm{p}} = 0.5$, and $\varepsilon_{\beta} = \varepsilon_{\mathrm{r}} = 0$, bottom row, D). In all cases, the situation modelled corresponds to newborns, and the trial is simulated on IGRA-negative cohorts. In all cases explored, the fraction of total TB cases that correspond to fast progressors remain above 94% (panel A). The fractions of trial realizations yield valid estimates of vaccine parameters that are above 99.9% for the vaccine based on ε_{p} and 77% for the one based on ε_{r} (panel B). Final distributions of the estimated parameters for both vaccines are largely unbiased with respect to the ground truth values, showing deviations that remain lower than 0.018 standard deviations in all examples tested (C,D).

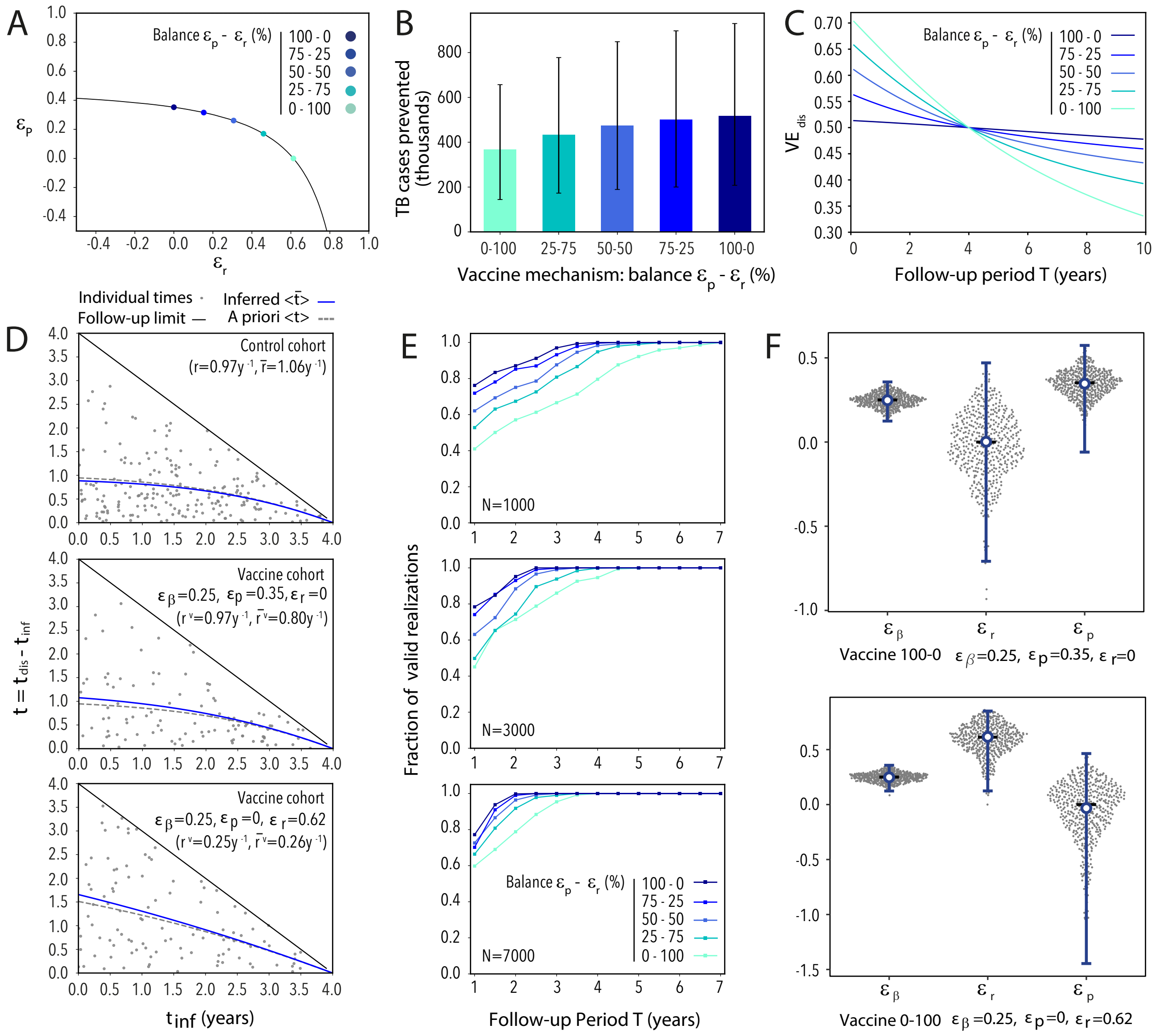

Supplementary figure 6. Characterization of vaccines conferring simultaneously POI and POD. These vaccines are equivalent to those of the main text, compatible with the same measurements of efficacy against disease (i.e. $\mathrm{VE_{dis}}(T = 4\ \mathrm{y})$) but incorporating efficacy against infection. A: Curve of values of (ε_p,ε_r) compatible with a measurement of $\mathrm{VE_{dis}} = 0.5$ after 4 years of follow-up (assuming $\varepsilon_\beta = 0.25$). We have marked 5 different points in this curve to be used in next examples. B: Predicted impacts obtained after introducing the 5 highlighted vaccines in a TB spreading model[5] in Ethiopia evaluated in the period 2025-2050. The relative difference between the first vaccine ($\varepsilon_\beta = 0.25, \varepsilon_\mathrm{p} = 0.35, \varepsilon_\mathrm{r} = 0$) and the last one ($\varepsilon_\beta = 0.25, \varepsilon_\mathrm{p} = 0, \varepsilon_\mathrm{r} = 0.62$) is 41% (35-49, 95% CI). C: Evolution of measurement of $\mathrm{VE_{dis}}$ for the 5 highlighted vaccines as a function of the follow-up period. D: Transition times for the control cohort, and the vaccine cohort for the two vaccines considered (balance 0-100 and 100-0). As in this analysis we are comparing equivalent vaccines to those of the main text (same $\mathrm{VE_{dis}}$ after 4 years), these vaccines have a smaller effect on the pathways against disease, so the number of transitions remains, approximately, the same; and our method is able to extract the rates of fast-progression. E. Probability density of the inferred parameters of the vaccines ($\varepsilon_\beta, \varepsilon_\mathrm{r}, \varepsilon_\mathrm{p}$), alongside the inferred parameters (with their respective CI) for two different vaccines: ε_p-based (top) and ε_r-based (bottom) (additionally to the protection against infection $\varepsilon_\beta = 0.25$). F: Fraction of correct realizations of a trial (i.e. realizations from which we obtain an epidemiologically plausible parametrization) as a function of the follow-up period, for three different cohort sizes and 5 different vaccines (the 5 vaccines remarked in panel A). Error bars (black bars in B and blue bars in F) represent the 95% confidence interval.

References

[1] Andrews, JR, Noubary, F, Walensky, RP, Cerda, R, Losina, E and Horsburgh, CR. Risk of progression to active tuberculosis following reinfection with Mycobacterium tuberculosis *Clin Infect Dis*, 54(6), 784-791, 10.1093/cid/cir951, 2012.

[2] Michele D Tameris, Mark Hatherill, Bernard S Landry, Thomas J Scriba, Margaret Ann Snowden, Stephen Lockhart, Jacqueline E Shea, J Bruce McClain, Gregory D Hussey, Willem A Hanekom, et al. Safety and efficacy of mva85a, a new tuberculosis vaccine, in infants previously vaccinated with bcg: a randomised, placebo-controlled phase 2b trial. *Lancet*, 381(9871):1021–1028, 10.1016/S0140-6736(13)60177-4, 2013.

[3] Peter R Cox. *Life tables*. Wiley Online Library, 1972.

[4] David Schoenfeld. Partial residuals for the proportional hazards regression model. *Biometrika*, 69(1):239–241, 10.2307/2335876 , 1982.

[5] Arregui S, Iglesias MJ, Samper S, Marinova D, Martin C, Sanz J and Moreno, Y. Data-driven model for the assessment of Mycobacterium tuberculosis transmission in evolving demographic structures. *Proc Natl Acad Sci U S A*, 115(14):E3238-E3245, 10.1073/pnas.1720606115, 2018.

[6] World Health Organization Tuberculosis Database, http://www.who.int/tb/country/en/index.html (accessed November 2016), 2016

[7] Kiti, Moses Chapa and Kinyanjui, Timothy Muiruri and Koech, Dorothy Chelagat and Munywoki, Patrick Kiio and Medley, Graham Francis and Nokes, David James, Quantifying age-related rates of social contact using diaries in a rural coastal population of Kenya, *PloS one*, 9(8), e104786, 10.1371/journal.pone.0104786, 2014.

[8] Melegaro, Alessia and Del Fava, Emanuele and Poletti, Piero and Merler, Stefano and Nyamukapa, Constance and Williams, John and Gregson, Simon and Manfredi, Piero Social Contact Structures and Time Use Patterns in the Manicaland Province of Zimbabwe, *PloS one*, 12 (1), e0170459, 10.1371/journal.pone.0170459, 2017.

[9] le Polain de Waroux, Olivier and Cohuet, Sandra and Ndazima, Donny and Kucharski, Adam and Juan-Giner, Aitana and Flasche, Stefan and Tumwesigye, Elioda and Arinaitwe, Rinah and Mwanga-Amumpaire, Juliet and Boum, Yap and others Characteristics Of Human Encounters And Social Mixing Patterns Relevant To Infectious Diseases Spread By Close Contact: A Survey In Southwest Uganda, *BMC Infect Dis*, 18(1), 1471-2334, 10.1186/s12879-018-3073-1, 2018.

[10] Arregui, S, Aleta, A, Sanz, J, and Moreno, Y. Projecting social contact matrices to different demographic structures, *PLoS Comput Biol*, 14(12), e1006638, 10.1371/journal.pcbi.1006638, (2018)

[11] Harris, R. C., Sumner, T., Knight, G. M., and White, R. G. Systematic review of mathematical models exploring the epidemiological impact of future TB vaccines. *Hum Vaccin Immunother*, 12(11), 2813-2832, 10.1080/21645515.2016.1205769, 2016.

[12] Mossong J, Hens N, Jit M, Beutels P, Auranen K, Mikolajczyk R, et. al. Social contacts and mixing patterns relevant to the spread of infectious diseases. *PLoS Med*;5(3):e74, 10.1371/journal.pmed.0050074, 2008.

[13] Population Division Database, http://esa.un.org/unpd/wpp/index.htm (accessed November 2016), 2016

[14] WHO. Tuberculosis database. *http://apps.who.int*, (accesed December 2017).

[15] Ahmet Soysal, Kerry A Millington, Mustafa Bakir, Davinder Dosanjh, Yasemin Aslan, Jonathan J Deeks, Serpil Efe, Imogen Staveley, Katie Ewer, and Ajit Lalvani. Effect of bcg vaccination on risk of mycobacterium tuberculosis infection in children with household tuberculosis contact: a prospective community-based study. *Lancet*, 366(9495):1443–1451, 10.1016/S0140-6736(05)67534-4, 2005.

[16] A Roy, M Eisenhut, RJ Harris, LC Rodrigues, S Sridhar, S Habermann, L Snell, P Mangtani, I Adetifa, A Lalvani, et al. Effect of bcg vaccination against mycobacterium tuberculosis infection in children: systematic review and meta-analysis. *BMJ*, 349:g4643, 10.1136/bmj.g4643, 2014.
