# Supplementary material for: Bridging the gap between efficacy trials and model-based impact evaluation for new tuberculosis vaccines
Source: Nat Commun. 2019 Nov 29;10:5457. doi: 10.1038/s41467-019-13387-9 (PMC6884451; doi:10.1038/s41467-019-13387-9)
Supplement: Supplementary file 2 — Reporting Summary [file 41467_2019_13387_MOESM2_ESM.pdf]

nature research

Corresponding author(s): Joaquín Sanz, Yamir Moreno.

Last updated by author(s): Oct 17, 2019

Reporting Summary

Nature Research wishes to improve the reproducibility of the work that we publish. This form provides structure for consistency and transparency in reporting. For further information on Nature Research policies, see Authors & Referees and the Editorial Policy Checklist .

Statistics

For all statistical analyses, confirm that the following items are present in the figure legend, table legend, main text, or Methods section.

n/a	Confirmed	
☐	☒	The exact sample size (n) for each experimental group/condition, given as a discrete number and unit of measurement
☒	☐	A statement on whether measurements were taken from distinct samples or whether the same sample was measured repeatedly
☐	☒	The statistical test(s) used AND whether they are one- or two-sided<br>*Only common tests should be described solely by name; describe more complex techniques in the Methods section.*
☒	☐	A description of all covariates tested
☐	☒	A description of any assumptions or corrections, such as tests of normality and adjustment for multiple comparisons
☐	☒	A full description of the statistical parameters including central tendency (e.g. means) or other basic estimates (e.g. regression coefficient) AND variation (e.g. standard deviation) or associated estimates of uncertainty (e.g. confidence intervals)
☒	☐	For null hypothesis testing, the test statistic (e.g. F, t, r) with confidence intervals, effect sizes, degrees of freedom and P value noted<br>*Give P values as exact values whenever suitable.*
☒	☐	For Bayesian analysis, information on the choice of priors and Markov chain Monte Carlo settings
☒	☐	For hierarchical and complex designs, identification of the appropriate level for tests and full reporting of outcomes
☐	☒	Estimates of effect sizes (e.g. Cohen's d, Pearson's r), indicating how they were calculated

Our web collection on statistics for biologists contains articles on many of the points above.

Software and code

Policy information about availability of computer code

Data collection	The data analyzed in this work was originated within in-silico simulations of clinical trials, as described in the text. The code needed to reproduce such simulations is available at: https://github.com/MarioTovarCalonge/NC_Tovar_Arregui_codes. As specified in the code availability statement, this repository includes algorithms written in Python (tested in version 3.6.7 2018-10-20), and R (tested in version 3.4.4 2018-03-15), with dependences to the following specific libraries: scipy, from Python, and bbmle [31] and OIsurv [28], from R. Scipy was used to implement the Brents numerical method used to solve numerically the mathematical constraints between vaccine mechanisms and efficacy readouts. bbmle to run the maximum likelihood models used to estimate within-cohort rates of transition to disease. OIsurv was used to run Cox regression methods.
Data analysis	In the same github repository, the scripts used to analyze the data originated in the simulations, and infer vaccine descriptions from them, is available as well.

For manuscripts utilizing custom algorithms or software that are central to the research but not yet described in published literature, software must be made available to editors/reviewers. We strongly encourage code deposition in a community repository (e.g. GitHub). See the Nature Research guidelines for submitting code & software for further information.

Data

Policy information about availability of data

All manuscripts must include a data availability statement. This statement should provide the following information, where applicable:

- Accession codes, unique identifiers, or web links for publicly available datasets
- A list of figures that have associated raw data
- A description of any restrictions on data availability

The data underlying the results presented in this study come from computational simulations that can be reproduced using the code available at: https://github.com/MarioTovarCalonge/NC_Tovar_Arregui_codes

Field-specific reporting

Please select the one below that is the best fit for your research. If you are not sure, read the appropriate sections before making your selection.

- [x] Life sciences
- [ ] Behavioural & social sciences
- [ ] Ecological, evolutionary & environmental sciences

For a reference copy of the document with all sections, see nature.com/documents/nr-reporting-summary-flat.pdf

Life sciences study design

All studies must disclose on these points even when the disclosure is negative.

Sample size	As a reference, we produced repetitions of n=500 simulated clinical trials of vaccine efficacy, from the different types of vaccines studied.
Data exclusions	Once the clinical trials were simulated, we applied a statistical inference strategy to obtain vaccine descriptors from the simulated data. IIn order to do that, as described in the manuscript, we first imposed a series of basic requisites (see Methods: a vaccine cannot delay fast-progression to disease to make it slower than slow progression, or modify probabilities of fast progression out from the interval [0,1]), to define meaningful ranges for the parameters, and excluded the simulations producing descriptors out from these ranges (see figure 2D: proportion of data excluded in different settings)
Replication	N/A
Randomization	N/A
Blinding	N/A

Reporting for specific materials, systems and methods

We require information from authors about some types of materials, experimental systems and methods used in many studies. Here, indicate whether each material, system or method listed is relevant to your study. If you are not sure if a list item applies to your research, read the appropriate section before selecting a response.

Materials & experimental systems

n/a	Involved in the study	
x		Antibodies
x		Eukaryotic cell lines
x		Palaeontology
x		Animals and other organisms
x		Human research participants
x		Clinical data

Methods

n/a	Involved in the study	
x		ChIP-seq
x		Flow cytometry
x		MRI-based neuroimaging
